# Review on two-dimensional material-based field-effect transistor biosensors: accomplishments, mechanisms, and perspectives

**DOI:** 10.1186/s12951-023-01898-z

**Published:** 2023-04-30

**Authors:** Shuo Chen, Yang Sun, Xiangyu Fan, Yazhe Xu, Shanshan Chen, Xinhao Zhang, Baoyuan Man, Cheng Yang, Jun Du

**Affiliations:** 1 School of Physics and Electronics, Shandong Normal University, Jinan, 250014 People’s Republic of China; 2 Beijing Key Laboratory for Bioengineering and Sensing Technology, School of Chemistry and Biological Engineering, University of Science and Technology, 30 Xueyuan Road, Haidian District, Beijing, 100083 People’s Republic of China

**Keywords:** Two-Dimensional Material, Field-effect transistor, Biosensor, Biomarker detection, Sensing application

## Abstract

Field-effect transistor (FET) is regarded as the most promising candidate for the next-generation biosensor, benefiting from the advantages of label-free, easy operation, low cost, easy integration, and direct detection of biomarkers in liquid environments. With the burgeoning advances in nanotechnology and biotechnology, researchers are trying to improve the sensitivity of FET biosensors and broaden their application scenarios from multiple strategies. In order to enable researchers to understand and apply FET biosensors deeply, focusing on the multidisciplinary technical details, the iteration and evolution of FET biosensors are reviewed from exploring the sensing mechanism in detecting biomolecules (research direction 1), the response signal type (research direction 2), the sensing performance optimization (research direction 3), and the integration strategy (research direction 4). Aiming at each research direction, forward perspectives and dialectical evaluations are summarized to enlighten rewarding investigations.

## Introduction

Field-effect transistor (FET) biosensors have attracted widespread attention in disease diagnosis, benefiting from their advantages of direct contact between gate and test solution, easy to form a solution detection environment, and the low working gate voltage (1–2 V) [1–3]. It is the most promising candidate for detecting disease-related biomarkers such as nucleic acids [4, 5], characteristic proteins [6, 7], and human secretions [8, 9].

The reviews summarizing the current breakthrough and development potential of FET biosensors from the aspect of nanomaterial science have been reported several times [10–12]. However, such reviews lack the discussion of multidisciplinary technical details, preventing researchers in this field from intuitively discovering the challenges behind the development of FET biosensors. Focusing on multidisciplinary technical details (including biomedical engineering, biophysics, analytical chemistry, and nanomaterial science), we summarized published research articles about FET biosensors in the past five years to point out the challenges and enhance the interdisciplinary understanding of readers. The iteration and evolution of FET biosensors were reviewed from exploring the sensing mechanism in detecting biomolecules (research direction 1), broadening the response signal type (research direction 2), optimizing the sensing performance (research direction 3), and promoting the intelligence and integration (research direction 4). Here, the progress and challenges of each research direction in the iteration and evolution process were analyzed and discussed.

For research direction 1, although increasing numbers of sensing mechanisms were developed, the reported sensing mechanisms can only explain the partly experimental results. Sometimes these sensing mechanisms are different in the same detection system. The Dirac point (indication signal) shifts in opposite directions in different reported works, which detect the same biomolecules [4, 5]. Researchers often sidestep this long-standing controversy by selecting the reported works that match the obtained experimental results to support their conclusion, which is obviously unreasonable and often confusing to readers. Lacking a universal sensing mechanism to explain all the experimental results is the main obstacle for FET biosensors.

For research direction 2, four types of the response signal have been explored to indicate the target molecules' detection. Different types of response signals have different sensitivities [13, 14]. However, for the specific sensing issue, researchers usually used only one type of response signal for biosensing, and thus the more sensitive performance of their proposed sensor may be lost by ignoring the detected results indicated by other types of response signals or that they haven't learned about these other signal types.

For research direction 3, various strategies based on novel nanotechnology and biotechnology to enhance the detection capability have been widely reported but not comprehensively summarized in technical detail. Currently, researchers have successfully optimized the sensing performance of FET biosensors in several ways, including sensing materials [15, 16], probes [17, 18], and signal amplification [19, 20]. However, for the specific issue, researchers usually focus on a specific strategy to improve sensitivity instead of applying a holistic approach that encompasses multiple strategies.

**Scheme 1:**
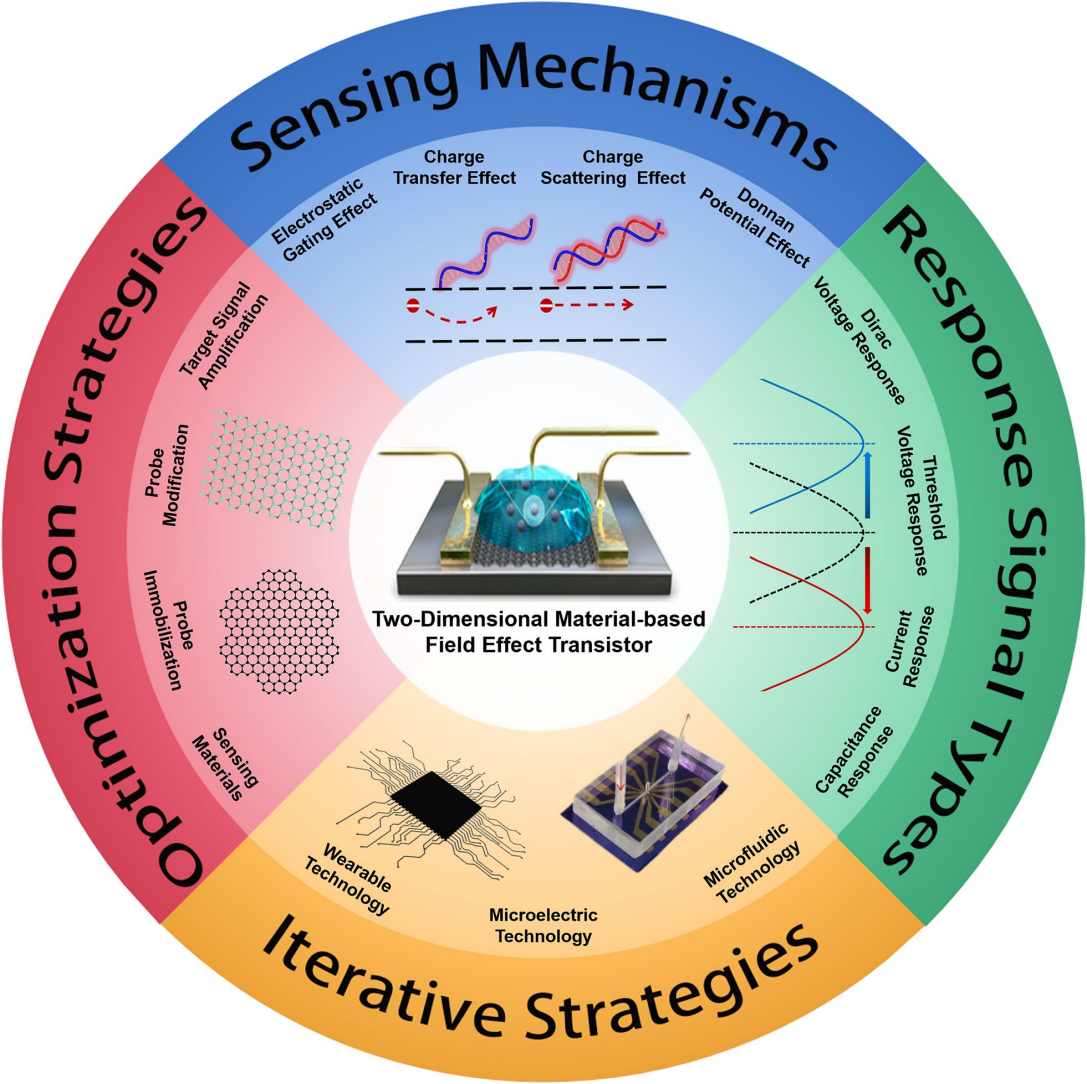
A brief introduction of two-dimensional material-based FET (2D material-based FET) on the sensing mechanisms, response signal types, optimization strategies, and iterative strategies. Reproduced with permission [30]. Copyright 2020, American Chemical Society. Reproduced with permission [5]. Copyright 2017, Nature Publishing Group

For research direction 4, researchers have combined other advanced technologies (microfluidics [21], microelectronics [22], wearable technology [23]) with FET biosensors and explored a variety of methods to promote the iterative development of FET biosensors, such as microfluidic chips with sample separation, purification, and quantitative injection [24, 25]; Intelligent detection platforms with the functions of signal identification, signal processing, and wireless transmission functions [26, 27]; Wearable biosensors with multiple application scenarios (on the skin surface or the eyeball) [28, 29]. A series of works have extensively promoted the process of intelligence and integration. FET with on-site detection and real-time monitoring functions has been well developed at the laboratory level. In the future, more optimization strategies such as human–machine interaction, cost reduction, and mass production need to be further developed and researched for commercial applications (see Scheme 1).

## Sensing mechanisms in detecting biomolecules

Various sensing mechanisms for FET biosensing have been proposed in the past ten years. However, the reported sensing mechanisms can only explain part of the experimental results, and a universal sensing mechanism still lacks. In order to inspire researchers to explore solutions, the progress and controversial points of existing sensing mechanisms are summarized.

### Electrostatic gating effect

Exogenously charged biomolecules induce sensing materials to generate opposite charges [39, 40]. In FET biosensing, the biomolecules-induced charges dope the sensing material to change its electrical properties, resulting in a detectable response signal [41, 42].

**Fig. 1:**
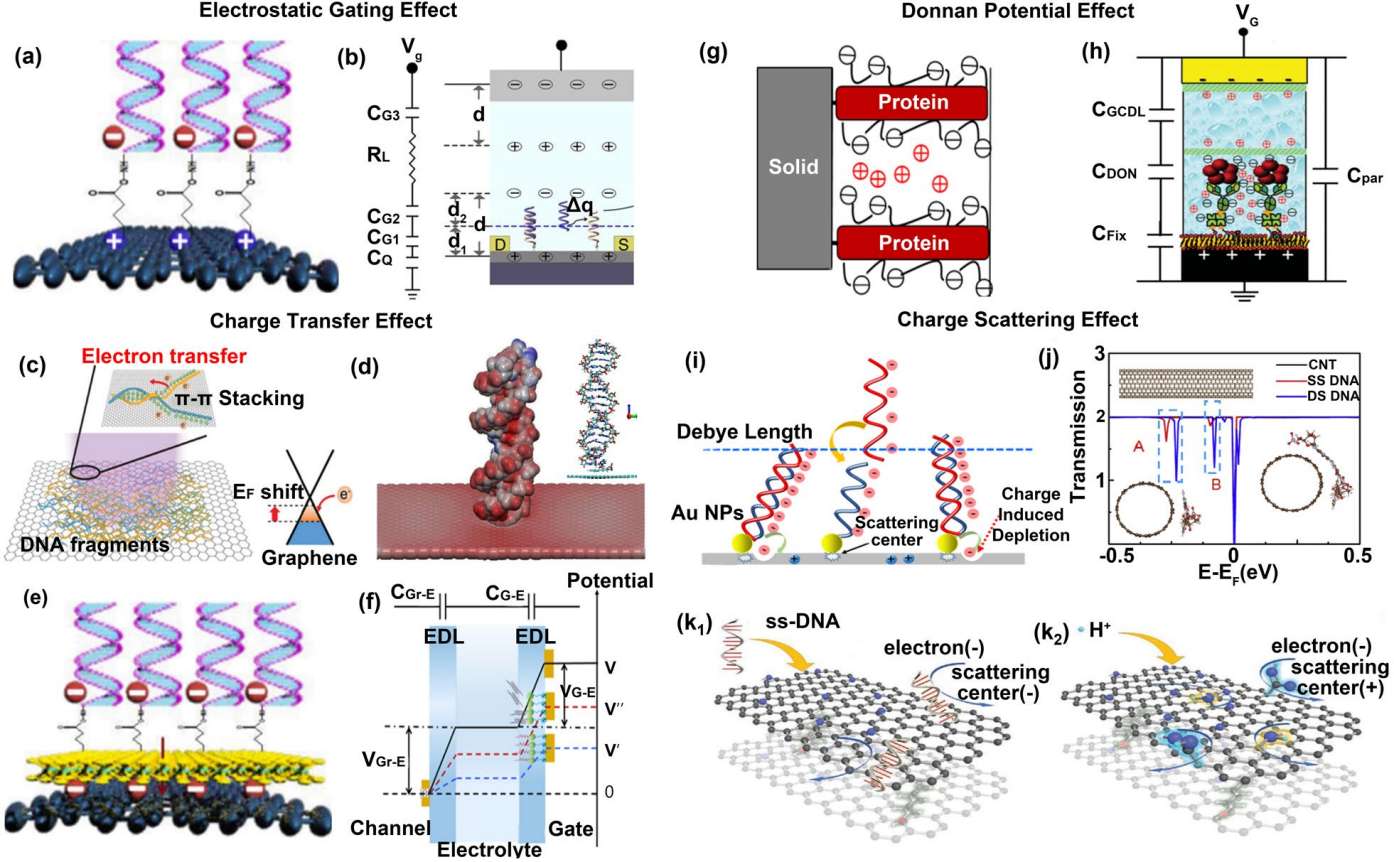
Sensing mechanisms in FET biomolecule detection. **a** and **b** Electrostatic gating effect and geometrical capacitance model. **c** Electron transfer between graphene and DNA. **d** Electrostatic potential simulation of graphene surface after binding double-stranded DNA. **e** Charge transfer between graphene and DNA induced by MoS_2_. **f** Potential modulation mechanism based on charge transfer. **g** and **h** Donnan potential-based potential distribution and capacitance model. **i** Electron scattering effect. **j** Transmission spectrum simulation of sensing material after binding DNA. **k** Charge scattering caused by adsorption of DNA (K1) and surface groups (K2) on graphene. (a,e) Reproduced with permission [31]. Copyright 2020, Elsevier Ltd. **b** Reproduced with permission [5]. Copyright 2017, Nature Publishing Group. **c** Reproduced with permission [32]. Copyright 2021, Royal Society of Chemistry. **d** Reproduced with permission [33]. Copyright 2021, Springer Netherlands. **f** Reproduced with permission [34]. Copyright 2022, American Chemical Society. **g**, **h** Reproduced with permission [35]. Copyright 2015, Wiley–VCH Verlag GmbH & Co. KGaA, Weinheim. **i** Reproduced with permission [36]. Copyright 2020, American Chemical Society. **j** Reproduced with permission [37]. Copyright 2019, Elsevier Ltd. **k** Reproduced with permission [38]. Copyright 2021, Wiley–VCH Verlag GmbH & Co. KGaA, Weinheim

Studies of FET biosensing show that the negative charges on the phosphate groups of DNA contribute P doping to graphene through the gating effect (Fig. 1a) [31], thereby changing its Fermi level and modulating its carrier density, resulting in a detectable response signal [43, 44]. A "geometrical" capacitance model was used to qualitatively explain the mechanism of how the carrier density (n) of sensing material is modulated by charged molecules through the electrostatic gating effect (Fig. 1b) [5]. Based on the "geometrical" capacitance model, the DNA density (${\uprho }$), the hybridization efficiency (HE), and binding affinity (K_A_) can be calculated by analyzing the changing carrier density. The "geometrical" capacitance represents the total capacitance of this FET sensor and can be described as Eq. (1):

1 $$\begin{array}{*{20}c} {{\text{C}} = \left( {\frac{1}{{{\text{C}}_{{{\text{G1}}}} }} + \frac{1}{{{\text{C}}_{{{\text{G2}}}} }} + \frac{1}{{{\text{C}}_{{{\text{G3}}}} }} + \frac{1}{{{\text{C}}_{Q} }}} \right)^{ - 1} } \\ \end{array}$$

where ${\text{C}}_{{{\text{G}}1}}$, ${\text{C}}_{{{\text{G}}2}}$ and ${\text{C}}_{{{\text{G}}3}}$ denote the capacitance between graphene and solution, the capacitance of the DNA to the solution, and the capacitance between Pt electrode and solution. Without bound charged molecules, the n is mainly modulated by the gate voltage (${\text{V}}_{{\text{G}}}$), as shown in Eq. (2) [45, 46].

2 $$n = \frac{{{\text{C}}_{{\text{g}}} \left( {{\text{V}}_{{\text{G}}} + {\text{V}}_{0} } \right)}}{{\text{q}}}$$

For the sensor bound with charged molecules, except for gate voltage, n is also modulated by negative charged molecules (${\text{V}}_{{{\text{molecules}}}}$), as shown in Eq. (3) [47].

3 $$\begin{array}{*{20}c} {n = \frac{{{\text{C}}_{{\text{g}}} }}{{\text{q}}}\left( {{\text{V}}_{{\text{G}}} + {\text{V}}_{0} } \right) + \frac{{{\text{C}}_{{\text{g}}} }}{{\text{q}}}{\text{V}}_{{\text{molecules }}} } \\ \end{array}$$

C_g_ is the gate capacitance per unit area. ${\text{V}}_{{\text{G}}}$ is gate voltage, ${\text{V}}_{0}$ is the natural voltage equivalent to the carrier inherent in the sensing material. So the modulation of the carrier density ($\Delta {\text{n}}$) by negative charged molecules is related to the change of ${\text{V}}_{{{\text{molecules}}}}$($\Delta {\text{V}}_{{{\text{molecules}}}}$), as shown in Eq. (4).

4 $$\Delta n = \frac{{{\text{C}}_{{\text{g}}} }}{{\text{q}}}\Delta {\text{V}}_{{{\text{molecules}}}}$$

The relation between the readable output ($\Delta {\text{V}}_{{{\text{cnp}}}}$) and the DNA density (${\uprho }$) can be described as Eq. (5) [48]:

5 $$\Delta {\text{V}}_{{{\text{cnp}}}} = \frac{{\Delta {\text{Q}}}}{{\text{C}}} = \frac{{{\text{Ne}}\rho {\text{S}}}}{{\text{C}}}$$

N is the number of bases in the added DNA, and S is the sensing area. So the probe density (${\uprho }_{{{\text{probe}}}}$) was calculated as Eq. (6a) and (6b) [5]:

6a $$\rho_{{{\text{probe}}}} = \frac{{\Delta {\text{V}}_{{{\text{cnp\_probe}}}} {\text{C}}}}{{{\text{N}}_{{{\text{probe}}}} {\text{eS}}}}$$

6b $$\begin{array}{*{20}c} {\Delta {\text{V}}_{{{\text{cnp}}\_{\text{probe}}}} = {\text{V}}_{{{\text{cnp}}\_{\text{probe}}}} - {\text{V}}_{{{\text{cnp}}\_{\text{PBASE}}}} } \\ \end{array}$$

The target density (${\uprho }_{{{\text{target}}}}$) was calculated as Eq. (7a) and (7b):

7a $$\rho_{{{\text{target}}}} = \frac{{\Delta {\text{V}}_{{{\text{cnp}}\_{\text{target}}}} {\text{C}}}}{{{\text{N}}_{{{\text{target}}}} {\text{eS}}}}$$

7b $$\begin{array}{*{20}c} {\Delta {\text{V}}_{{{\text{cnp}}\_{\text{target}}}} = {\text{V}}_{{{\text{cnp}}\_{\text{target}}}} - {\text{V}}_{{{\text{cnp}}\_{\text{probe}}}} } \\ \end{array}$$

Combining the above Equations, the HE was quantitatively calculated by Eq. (8):

8 $$HE = \frac{{\rho_{{{\text{target}}}} }}{{\rho_{{{\text{probe}}}} }} \times 100\% = \frac{{\Delta {\text{V}}_{{{\text{cnp}}\_{\text{target}}}} }}{{\Delta {\text{V}}_{{{\text{cnp}}\_{\text{probe}}}} }} \times 100\%$$

${\text{K}}_{{\text{A}}}$ can also be calculated by the concentration-dependent maximal $\Delta {\text{V}}_{{{\text{cnp}}}}$ ($\Delta {\text{V}}_{{{\text{max cnp}}\_{\text{target}}}}$) and the readable output of the target DNA $\left( {\Delta {\text{V}}_{{{\text{cnp}}\_{\text{target}}}} } \right)$ as shown in Eq. (9).

9 $$\frac{{\Delta {\text{V}}_{{{\text{cnp}}\_{\text{target}}}} }}{{\Delta {\text{V}}_{{{\text{max cnp}}\_{\text{target}}}} }} = \frac{{{\text{K}}_{{\text{A}}} \left[ {\text{A}} \right]}}{{{\text{K}}_{{\text{A}}} \left[ {\text{A}} \right] + 1}}$$

[A] is the solution concentration of the analyte.

In the above-reported works of DNA detection by using graphene FET, exogenous DNA contributes P doping to graphene through the gating effect, which reduces the Fermi level of graphene and makes the Dirac voltage shift towards the positive gate voltage. However, in other reported works that detected DNA using graphene FET [49–51], Dirac voltage shifts towards the negative gate voltage. This shows that it is difficult to explain all experimental results based on the electrostatic gating effect, and the details will be described in “Dirac voltage response” section.

### Charge transfer effect

The charges interact-transfer between biomolecules and sensing materials [52]. In FET biosensing, the transfer of charge causes an increase or decrease in the carrier density of sensing material, resulting in a detectable response signal [41, 53].

Reported works proposed that the aromatic ring of the base of DNA acts as the electron donor to dope graphene, modulating the carrier concentration of graphene and resulting in a detectable response signal [54, 55]. Ai et al. proposed that the electrons carried on the aromatic ring of the base of single-stranded DNA can be transferred to P-type graphene, decreasing the hole concentration of graphene by studying a DNA origami structure-based FET (Fig. 1c) [32]. Sheida Bagherzadeh‑Nobari’s work based on DFT simulation shows that electron-rich aromatic rings of the base of single-stranded DNA are placed horizontally on the graphene surface by π-π stacking, making electrons transfer from the aromatic ring of the base to graphene and increasing the electron concentration of graphene. The simulation results also show that a further increase of graphene's electron density occurred by accepting more electrons when the double-stranded DNA is immobilized on the graphene surface (Fig. 1d) [33].

Furthermore, our team proposed a competitive mechanism between the gating effect and the charge transfer effect [31]. We believed that the signals detected during sensing result from the superposition of multiple mechanisms rather than being determined by a single mechanism. The dominance of the two effects determines whether the carrier concentration increases or decreases. Here, the polar MoS_2_ interlayer closes the distance between them by polarizing DNA, allowing DNA to modulate the carrier concentration of the sensing material through the charge transfer effect (Fig. 1e). When the MoS_2_ density on the graphene surface is low, the electrostatic gating effect plays a major role in modulating the carrier concentration of graphene. When the MoS_2_ density on the graphene surface is high, the charge transfer effect plays a larger role in modulating the carrier concentration of graphene than the electrostatic gating effect.

To prove that DNA can modulate the carrier density of the sensing material through the charge transfer effect, Deng et al. developed a capacitive model to qualitatively explain the mechanism (Fig. 1f) [34]. Here, a relation between the readable output current (${\text{I}}_{{{\text{ds}}}}$) and the gate potential change ($\Delta {\text{V}}^{{{\text{eff}}}}$) was defined, as shown in Eq. (10)–(13) [34, 56, 57].

10 $${\text{I}}_{{{\text{ds}}}} = \frac{{\text{W}}}{{\text{L}}}\mu {\text{C}}\left| {{\text{V}}^{{{\text{eff}}}} } \right. - {\text{V}}_{{{\text{Dirac}}}} - \left. {\frac{{{\text{V}}_{{{\text{ds}}}} }}{2}} \right|$$

11 $${\text{V}}^{{{\text{eff}}}} = {\text{V}}_{{{\text{GS}}}} - \Delta {\text{V}}^{{{\text{eff}}}}$$

12 $${\text{V}}_{{{\text{GS}}}} = {\text{V}}_{{{\text{G\_E}}}} + {\text{V}}_{{{\text{Gr\_E}}}}$$

13 $${\text{C}} = \left( {\frac{1}{{{\text{C}}_{{{\text{G\_E}}}} }} + \frac{1}{{{\text{C}}_{{{\text{Gr\_E}}}} }}} \right)^{ - 1}$$

${\text{V}}_{{{\text{G}}\_{\text{E}}}}$ is the potential between the gate electrode and the solution, ${\text{V}}_{{{\text{Gr}}\_{\text{E}}}}$ is the potential between the graphene and the solution, ${\text{V}}_{{{\text{Dirac}}}}$ is the Dirac voltage of the graphene, ${\text{V}}_{{{\text{ds}}}}$ is the potential between the source and drain, ${\text{V}}_{{{\text{GS}}}}$ is the gate voltage, ${\upmu }$ is the carrier mobility of graphene, ${\text{C}}$ is the total capacitance of graphene FET, ${\text{W}}$ is the channel width, ${\text{L}}$ is the channel length, and ${\text{V}}^{{{\text{eff}}}}$ is the effective gate voltage. Specifically, when DNA duplexes are formed on the gate (graphene) surface, the gate potential change ($\Delta {\text{V}}^{{{\text{eff}}}}$) caused by DNA through the charge transfer effect can be described as Eq. (14):

14 $$\Delta {\text{V}}^{{{\text{eff}}}} = \frac{{\Delta {\text{Q}}}}{{\text{C}}}$$

$\Delta {\text{Q}}$ is the charge change at the gate surface due to the modulation of DNA. The negative charges carried on the probe DNA can be transferred to the gate surface, which is equivalent to applying a negative voltage to the gate(${\text{V}}_{{{\text{GS}}}}$ decrease, black line → blue line), making the Dirac voltage shift towards the negative gate voltage and causing ${\text{V}}^{{{\text{eff}}}}$ and ${\text{I}}_{{{\text{ds}}}}$ decrease. Double-stranded DNA formed by the binding of target DNA and probe DNA can be detached from the gate surface, which is equivalent to applying a positive voltage to the gate (${\text{V}}_{{{\text{GS}}}}$ increase, blue line → red line), making the Dirac voltage shift towards the positive gate voltage and causing ${\text{V}}^{{{\text{eff}}}}$ and ${\text{I}}_{{{\text{ds}}}}$ increase.

However, some studies based on DFT proposed that the frontier molecular orbital energy level of nucleosides (including the highest occupied and lowest unoccupied molecular orbitals) is far away from the Fermi level of graphene [43, 58, 59], which means that the charge transfer between nucleosides and graphene is negligible. Therefore, whether there is charge transfer between graphene and nucleosides is controversial, and the sensing mechanism between graphene and nucleosides still needs further study.

### Donnan potential effect

The gating effect only explains why biomolecules within the Debye length can be detected. However, many works of protein or bacterial detection based on FET shows that biomolecules beyond the Debye length can be successfully detected [60], which indicates the gating effect cannot explain all the experimental results. The results where biomolecules beyond the Debye length can be detected contribute to the Donnan potential effect (Fig. 1g) [35].

The phase interface between the biomolecular layer and the bulk solution forms a semi-permeable membrane [61]. Since the charged molecules in the biomolecular layer cannot penetrate the semipermeable membrane, an inhomogeneous electric field is generated on two sides of the membrane, resulting in the potential difference (Donnan potential, Fig. 1h) [35]. Hajian et al. proposed that the biomolecular layer composed of charged molecules (such as proteins and nucleic acids) leads to the difference of salt ion concentration between bulk solutions outside the membrane (${\text{c}}_{{\text{s}}}$) and the biomolecular layer inside the membrane (${\text{c}}_{{\text{x}}}$), resulting in the Donnan potential ($\Delta \varphi_{{\text{D}}}$) [2, 3]. Any charges or dipoles resulting in net charges within the biomolecular layer require additional accumulation of counter-ions within the layer to maintain charge neutrality. So when charged biomolecules are detached or adsorbed from the sensing material surface, the salt ion concentration of the biomolecular layer changes, resulting in the change of Donnan potential, modulating the source-drain current (${\text{I}}_{{{\text{ds}}}}$), as shown in Eqs. (15) and (16) [61, 62].

15 $$\Delta \varphi_{{\text{D}}} =\Phi _{{{\text{th}}}} ln\frac{{\left( {\sqrt {4{\text{c}}_{s}^{2} + {\text{c}}_{{\text{x}}}^{2} + {\text{c}}_{{\text{x}}} } } \right)}}{{2{\text{c}}_{{\text{s}}} }}\,$$

16 $${\text{I}}_{{{\text{ds}}}} \approx \frac{{\text{W}}}{{\text{L}}}\mu {\text{CV}}_{{{\text{ds}}}} \left( {{\text{V}}_{0} - {\text{V}}_{{\text{g}}} + 2.3\Phi_{{{\text{th}}}} \alpha \Delta {\text{pH}} + \left( {1 - \alpha } \right)\Delta \varphi_{{\text{D}}} } \right)$$

${\upmu }$, ${\text{V}}_{{{\text{ds}}}}$, ${\text{V}}_{0}$, ${\text{V}}_{{\text{g}}}$, ${\text{W}}$, ${\text{L}}$, ${\upalpha }$, ${\Phi }_{{{\text{th}}}}$,${\text{ C}}$ and $\Delta {\text{pH}}$ are the charge carrier mobility, the source-drain voltage, the Dirac voltage, the gate voltage, channel width, channel length, the surface pH sensitivity factor, the thermal voltage, the total capacitance of graphene FET, and pH shift from a neutral surface to produce the equivalent gate voltage. ${\text{C}}$ is the series connection of the graphene quantum capacitance (${\text{C}}_{{\text{G}}}$), the electric double-layer capacitance (${\text{C}}_{{{\text{EDL}}}}$) and the Donnan capacitance (${\text{C}}_{{{\text{Donnan}}}}$), as shown in Eq. (17).

17 $${\text{C}} = \left( {\frac{1}{{{\text{C}}_{{\text{G}}} }} + \frac{1}{{{\text{C}}_{{{\text{EDL}}}} }} + \frac{1}{{{\text{C}}_{{{\text{Donnan}}}} }}} \right)^{ - 1}$$

The biomolecular layer is a necessary condition for forming the Donnan potential. The reported work on Donnan potential effects in biosensing only assumes the existence of a biomolecular layer. The experimental results are perfectly explained based on this hypothesis, but whether the biomolecular layer exists has not been experimentally proven and will be discussed further.

### Charge scattering effect

The periodic potential field inside the sensing material is disrupted by the charged biomolecules immobilized on the surface of the sensing material, which results in constant changes in the magnitude and direction of the carrier velocity [63]. Liang et al. proposed that the charged biomolecules on the surface of the sensing material can hinder the transport of carriers through the local carrier scattering effect (Fig. 1i) [36], thereby reducing the conductivity of the sensing material and causing the negative response current.

The DFT-based simulation results show that nucleic acids or other softly charged molecules immobilized on the surface of the sensing material can act as short-range scattering centers, reducing the local carrier concentration and carrier mobility, thereby causing a negative current response [37]. The depth of the valley peak in the transmission spectra represents the degree of weakening in the conductivity of the sensing material. As shown in Fig. 1j, the peaks in the wireframes labeled A and B indicate that the valleys caused by double-stranded DNA (DSDNA, blue line) are deeper than those caused by single-stranded DNA (SSDNA, red line), which indicates the ability of DSDNA to weaken the conductivity of sensing material was higher than that of SSDNA. The valley peaks caused by SSDNA move to the Fermi energy after DNA hybridization (the red line moves to the blue line), indicating that the conductivity of the sensing material is further weakened. Huang et al. proposed a graphene oxide-based FET to detect protonated carboxyl groups, protonated hydroxyl groups (Fig. 1k1), and single-stranded DNA (Fig. 1k2) [38]. The scattering centers formed by the absorption of these charged biomolecules to sensing material dramatically decrease the carrier mobility of sensing material, generating the response signal.

Compared with the other three reported effects, the experimental results based on the charge scattering effect are consistent in various reports, and no controversial points have been found so far.

## Response signal types of FET

In the past ten years, various types of response signals for FET biosensing have been proposed, but the relation between the response signal and the sensing mechanism was not summarized. In order to help researchers better understand the relation between them, the existing types of response signals corresponding to sensing mechanisms were summarized, and the corresponding mechanism was explained. Finally, the advantages and disadvantages of various response signals are discussed.

### Voltage response

#### Dirac voltage response

**Fig. 2:**
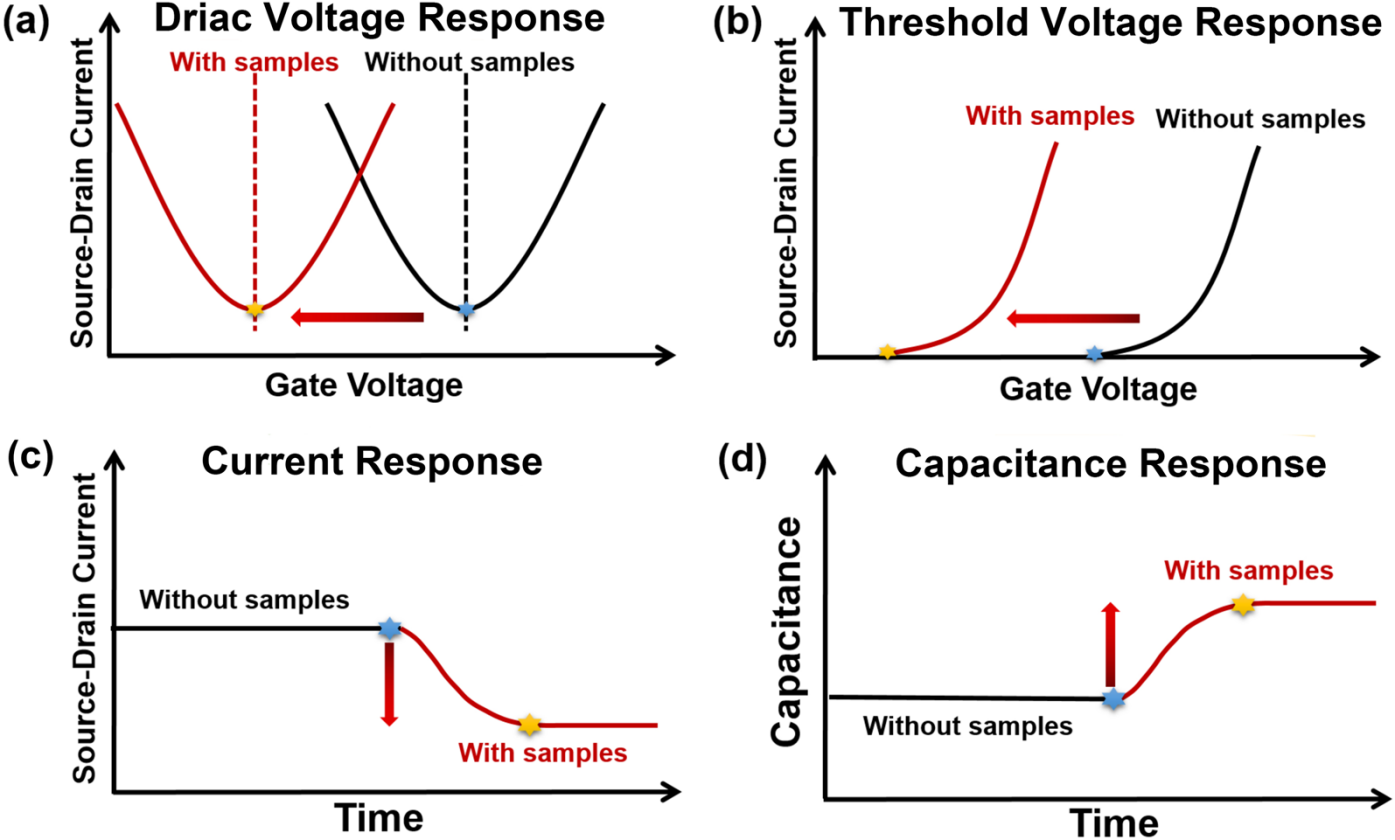
Response signal types of FET in detecting biomolecules. **a** Dirac voltage response of graphene FET. **b** Threshold voltage response of MX_2_ FET. **c** Current response. **d** Capacitance response

The exogenous hole or electron doping of biomolecules changes the Dirac voltage of graphene (Fig. 2a) [64, 65]. Based on this, the movement of Dirac voltage has been widely selected as an indication signal for graphene FET biosensing, as shown in Table 1.

**Table 1:** Statistics on the Dirac voltage response in graphene-based FET

Sensing materials	Functionalization methods	Probe types	Probe structure	Target biomarkers	Detection environment	LOD	References number	Year
CVD-Graphene	PBASE	DNA	Single-stranded	20-mer DNA	0.01 × PBS	10 pM	[5]	2017
CVD-Graphene	PBASE	DNA	Single-stranded	15-mer DNA	0.01 × PBS	1 nM	[66]	2018
CVD-Graphene	PBASE	DNA	Single-stranded	24-mer DNA	10 mM PBS	25 aM	[67]	2019
CVD-Graphene	PBASE	DNA	Single-stranded	19-mer DNA	1 nM TE	1 nM	[49]	2018
CVD-Graphene	PBASE	DNA	Hairpin	21-mer DNA	5 × SSC	5 fM	[68]	2018
CVD-Graphene	Physically adsorb	DNA	Single-stranded	20-mer DNA	0.10 mol/L PBS (pH 7.2)	5 zM	[69]	2020
CVD-Graphene	PBASE	DNA	Tetrahedral structure	28-mer RNA	Artificial saliva	0.02 copy/μL	[50]	2021
CVD-Graphene	SLB	Gangliosi	–	Antigen	PBS	12.5 nM	[70]	2018
CVD-Graphene	PBASE	DNA	Single-stranded	Antigen	0.01 × PBS	2.6 pM	[71]	2020
CVD-Graphene	CQDs	Antibody	Y-type structure	Antigen	0.001 × PBS	100 particles/μL	[72]	2021
CVD-Graphene	CQDs	DNA	Single-stranded	28-mer DNA	PBS	1 aM	[34]	2022
rGO	PtNPs	Antibody	Y-type structure	Antigen	0.001 × PBS	100 fM	[73]	2017
rGO	Glutaraldehyde cross-linking	RNA	Single-stranded	Antigen	10 mM PBS	1 pg/mL	[74]	2020
rGO	PBASE	Antibody	Y-type structure	Antigen	10 μM PBS	1 pg/mL	[75]	2020
B/N co-doped GO	Physically adsorb	Antibody	Y-type structure	Antigen	1 × PBS	10 aM	[76]	2021
Mxenes/graphene	APTES	Antibody	Y-type structure	Antigen	1 × PBS	1 fg/mL	[77]	2021
CVD-Graphene	PBASE	DNA	Single-stranded	15-mer RNA	0.1 × PBS	0.1 fM	[78]	2018
CVD-Graphene	PBASE	DNA	Tweezers	30-mer DNA	-	100 pM	[79]	2018
CVD-Graphene	PBASE	DNA	Single-stranded	Antigen	1 × PBS	26 pM	[80]	2018
CVD-Graphene	PBASE	DNA	Single-stranded	Antigen	1 × PBS	5 pM	[81]	2019
CVD-Graphene	PBASE	DNA	Single-stranded	Antigen	1 × PBS	139 fM	[82]	2019
CVD-Graphene	PBASE	DNA	Single-stranded	Antigen	1 × PBS	12 pM	[83]	2019
CVD-Graphene	PBASE	DNA	Single-stranded	22-mer DNA	1 × PBS	2 aM	[4]	2020
CVD-Graphene	PBASE	DNA	Single-stranded	30-mer DNA	PBS	10 aM	[84]	2021
CVD-Graphene	Physically adsorb	DNA	Single-stranded	Antigen	1 × PBS	1 μM	[85]	2018
CVD-Graphene	Physically adsorb	DNA	Single-stranded	30-mer DNA	1 × PBS	1 nM	[86]	2019
CVD-Graphene	PBASE	DNA	Y-type structure	28-mer RNA	0.1 × PBS	0.03 copy/μL	[87]	2021
CVD-Graphene	PBASE	DNA	Tetrahedral structure	mer DNA,	1 × TM	0.01 copy/μL	[1]	2022
CVD-Graphene	PBASE	Antibody	Y-type structure	Antigen	Artificial saliva	0.173 copy/μL	[88]	2021
CVD-Graphene	PBASE	Aptamer	Single-stranded	Antigen	0.01 × PBS	47 pM	[89]	2018
CVD-Graphene	PBASE	Antibody	Y-type structure	Antigen	PBS	1 fg/mL	[30]	2020
CVD-Graphene	PBASE	Antibody	Y-type structure	Antigen	TE	47.8 aM	[90]	2019
CVD-Graphene	PBASE	Antibody	Y-type structure	Antigen	Serum	2.6 aM	[91]	2021
CVD-Graphene	AuNPs	DNA	Single-stranded	20/21/34-mer DNA	0.01 × PBS	15 aM	[92]	2020
CVD-Graphene	PLL	DNA	Single-stranded	20/48-mer RNA	1 × PBS	1 fM	[93]	2022
CVD-Graphene	Glutaraldehyde cross-linking	DNA	Single-stranded	24-mer DNA	PBS	1 nM	[94]	2021
CVD-Graphene	Physically adsorb	DNA	Single-stranded	20-mer RNA	1 × PBS	10 fM	[95]	2020
CVD-Graphene	Nafion	DNA	Single-stranded	Antigen	1 × PBS	740 fM	[96]	2021
CVD-Graphene	PBASE	PNA	Single-stranded	15-mer RNA	1 × PBS	0.1 aM	[97]	2020
MoS_2_/graphene	PBASE	DNA	Single-stranded	15-mer DNA	1 × PBS	10 aM	[31]	2020
rGO	AuNPs	Antibody	Single-stranded	Antigen	0.1 × PBS	84 particles/μL	[65]	2020
rGO	PBASE	Antibody	Y-type structure	Antigen	10 mM PBS	222 fM	[75]	2020
rGO	Physically adsorb	Antibody	Y-type structure	Antigen	PBS	2.4 pg/mL	[98]	2018
rGO	AuNPs	PMO	Single-stranded	19-mer RNA	0.01 × PBS	0.29 fM	[99]	2021

Purwidyantri et al. used the Dirac voltage as a P-type graphene-based FET response signal to detect DNA hybridization [64]. They proposed that negative charges fixed on the graphene surface increase the hole density of P-type graphene through the local gating effect, resulting in a shift of Dirac voltage. Gao et al. also used the Dirac voltage as an indication signal of N-type graphene-based FET to detect DNA-miRNA hybridization [95]. In their work, the negative charges carried on the phosphate groups of probe DNA that are absorbed on graphene by π-π stacking make the electron density of N-type graphene increase through the charge transfer effect, resulting in the movement of Dirac voltage. After double helix formation between probe DNA and target miRNA, the electron density of N-type graphene further increases.

As shown in Table 1, using the shift of Dirac voltage as a response signal to detect trace biomolecules, the sensitivity has approached the level of single-molecule detection, and the detection limit is 0.1 aM.

#### Threshold voltage response

In MoS_2_ FET biosensing, the doping of exogenous biomolecules to MoS_2_ causes the change of threshold voltage (Fig. 2b), making the threshold voltage widely selected as the response signal [100]. Lee et al. used the threshold voltage as a response signal of N-type MoS_2_-based FET to detect DNA hybridization [101]. They proposed that the negative charges carried on the phosphate groups of probe DNA that absorbed on MoS_2_ by π-π stacking, make the electron density of N-type MoS_2_ decrease through the gating effect, resulting in the shift of threshold voltage. A layer of Mo atoms in the middle and S atoms on each side forms the sandwich structure of single-layer MoS2. The edges and defects of MoS_2_ are usually negatively charged due to the lone pair electrons of S atoms [102, 103]. After double helix formation between probe DNA and target DNA, the formed double-stranded makes the aromatic ring of the base be hidden within the double helix, resulting in the disappearance of π–π stacking. The repulsion between the negatively charged MoS_2_ and the negatively charged double-stranded DNA leads to the desorption of the double-stranded DNA from the MoS_2_ surface. This increases the electron density of MoS_2_, resulting in a shift in the threshold voltage. Through the mechanism that the electron density of MoS_2_ decreases first and then increases, the target molecule was successfully detected by the threshold voltage response. Ma et al. used the threshold voltage as a response signal of N-type MoS_2_ FET to detect ConA [104]. Here, the positive charges carried on ConA increase the electron density of N-type MoS_2_ through the gating effect, resulting in the threshold voltage shift. The above works indicate that the threshold voltage response can judge the detection results in MoS_2_-based FET biosensing.

At present, the threshold voltage as a response signal to detect biomolecules has only been reported by MoS_2_-based FET, which indicates other MX_2_-based FETs to detect biomolecules are also promising to use threshold voltage as a response signal, such as WS_2_ FET [105], WSe_2_ FET [106] and ReS_2_ FET [107]. Using the change of threshold voltage as a response signal to detect trace biomolecules, the sensitivity needs to be further explored and developed, and the current limit of detection is 10 fM.

### Current response

Except for the voltage response, the change of source-drain current (Fig. 2c) is also widely used as a response signal in FET biosensing [30, 108], as shown in Table 2.

**Table 2:** Statistics on the current response in graphene/MX_2_-based FET

Sensing materials	Functionalization methods	Probe types	Probe structure	Target biomarkers	Detection environment	LOD	Reference number	Year
CVD-Graphene	PBASE	RNA-Cas9	–	20-mer DNA	2 mM MgCl_2_	1.7 fM	[3]	2019
CVD-Graphene	CQDs	DNA	Single-stranded	28-mer DNA	PBS	1 aM	[34]	2022
CVD-Graphene	PBASE	Antibody	Single-stranded	Antigen	1 × PBS	139 fM	[82]	2019
CVD-Graphene	AuNPs	Antibody	Y-type structure	Antigen	PBS	0.4 pM	[108]	2020
CVD-Graphene	Physically adsorb	Antibody	Y-type structure	Antigen	1 × PBS	10 nM	[109]	2018
CVD-Graphene	EDC + NHS	Antibody	Y-type structure	Antigen	0.1 mM PBS	56 fM	[110]	2019
MoS_2_	Physically adsorb	Antibody	Y-type structure	Antigen	PBS	1 pM	[19]	2019
MoS_2_	AuNPs	DNA	Tetrahedral	Antigen	PBS	1 fg/mL	[111]	2021
MoS_2_	APTES	Antibody	Y-type structure	Antigen	PBS	10^–9^ g/L	[112]	2021
MoS_2_	Physically adsorb	RNA	Single-stranded	30-mer RNA	0.01 M PBS	0.1 fM	[13]	2018
WSe_2_	MUA	Antibody	Y-type structure	Antigen	0.01 × PBS	25 fg/μL	[106]	2021
WSe_2_	APTES	Antibody	Y-type structure	Antigen	PBS	10 fg/ml	[113]	2021
rGO	PBASE	RNA	Single-stranded	Antigen	0.1 M PBS	1.75 nM	[114]	2021
rGO	Electrostatic interaction	Urease	–	Arginase	10 mM KCl	10 μM	[8]	2018
CVD-Graphene	PBASE	DNA	Y-type structure	28-mer RNA	0.1 × PBS	0.3 aM	[87]	2021
CVD-Graphene	PBASE	DNA	Tetrahedral structure	28-mer RNA	Artificial saliva	0.2 aM	[50]	2021
CVD-Graphene	PBASE	DNA	Single-stranded	15-mer DNA	PBS	1 aM	[47]	2021
CVD-Graphene	PBASE	DNA	Tetrahedral structure	29-mer DNA	1 × TM	0.1 aM	[1]	2022
CVD-Graphene	PBASE	Antibody	Y-type structure	Antigen	0.1 × PBS	0.74 nM	[115]	2022
CVD-Graphene	PBASE	Antibody	Y-type structure	Antigen	PBS	0.37 pM	[116]	2020
CVD-Graphene	PBASE	Antibody	Y-type structure	Antigen	Serum	2.6 aM	[91]	2021
CVD-Graphene	PBASE	Antibody	Y-type structure	Antigen	Artificial saliva	1 aM	[88]	2021
CVD-Graphene	Physically adsorb	Antibody	Y-type structure	Antigen	1 × PBS	25 aM	[117]	2021
CVD-Graphene	Physically adsorb	Antibody	Y-type structure	Antigen	PBS	10 fg/mL	[118]	2020
Exfoliated-Graphene	EDC + NHS	Antibody	Y-type structure	Antigen	50 mM PBS	10 fg/mL	[119]	2019
MoS_2_	PBASE	PMO	Single-stranded	22-mer DNA	0.5 × PBS	6 fM	[16]	2018
MoS_2_	PBASE	Antibody	Y-type structure	Antigen	1 × PBS	100 fg/mL	[120]	2019
MoS_2_	AuNPs	DNA	Single-stranded	30-mer DNA	0.1 × PBS	10 aM	[15]	2019
MoS_2_	AuNPs	Antibody	Y-type structure	Antigen	0.1 × PBS	105 nM	[104]	2021
MoS_2_	APTES	DNA	Single-stranded	Cortisol	PBS	1 ag/mL	[121]	2021
MoS_2_	APTES	Antibody	Y-type structure	Antigen	PBS	10^–9^ μg/μL	[122]	2022
rGO	PtNPs	Antibody	Y-type structure	Antigen	0.001 × PBS	100 fM	[73]	2017
rGO	Physically adsorb	DNA	Single-stranded	48-mer DNA	1 × PBS	5 pM	[123]	2017
rGO	AuNPs	PMO	Single-stranded	19-mer RNA	0.01 × PBS	0.29 fM	[99]	2021
WS_2_	Physically adsorb	DNA	Single-stranded	18-mer DNA	0.1 × PBS	3 aM	[105]	2022

Timing reading of the current changes of FETs according to the reaction time of target molecules and probe molecules is a widely reported method to obtain response signals [31, 124]. The current response is more widely selected as the response signal than the threshold voltage response in MoS_2_-based FET biosensing. Mei et al. used the source-drain current as a response signal of MoS_2_-based FET to detect DNA hybridization [16]. In their work, charged biomolecules absorbed on the MoS_2_ surface decrease the carrier mobility of sensing material through the charge scattering effect, resulting in the change of source-drain current. Majd et al. used the source-drain current as a response signal of N-type MoS_2_-based FET to detect miRNA [13]. Here, after double helix formation between probe DNA and target miRNA, the formed double-stranded make aromatic rings of the base hidden within the double helix, resulting in the disappearance of π-π stacking. The repulsion between the negatively charged MoS_2_ and the negatively charged DNA-miRNA causes the desorption of the DNA-miRNA from the MoS_2_ surface. This increases the electron density of MoS_2_, changing the source-drain current. The sensing mechanism of SamiraMansouri Majd's work [13] for detecting miRNA is consistent with that of Doo-Won Lee's work [101] for detecting DNA. The current response instead of the threshold voltage response was used to judge the detection results in Samira Mansouri Majd's work. However, the detection limit (30 aM) in SamiraMansouri Majd's work is lower than that of 10 fM in Doo-Won Lee’s work, indicating that the current response is more sensitive than the threshold voltage response.

In addition, real-time reading of the source-drain current of FET is also a widely selected method to obtain the response signal [125, 126]. Wei’s team used the real-time change of source-drain current as a P-type graphene-based FET response signal to detect SARS-COV-2 RNA [50]. In their work, negative charges transfer from RNA to the graphene surface, decreasing the hole density of P-type graphene and resulting in the change of source-drain current. Nekrasov et al. also used the real-time change of source-drain current as an N-type graphene-based FET response signal to detect ochratoxin A [126]. Here, negative charges absorbed on the graphene surface decrease the electron density of sensing material through the gating effect, resulting in the change of source-drain current.

### Capacitance response

Recently, the change in total capacitance has been used as the response signal in FET biosensing (Fig. 2d). Aran’s team used the capacitance change as a response signal of graphene-based FET to detect DNA [2, 3]. Here, a biomolecular layer composed of charged molecules was formed on the graphene surface. The total capacitance of FET was modulated by the charged molecules adsorbed on the graphene surface through the Donnan potential effect. ${\text{C response}}$ was defined as the percentage change in the average of all slope values with biomolecules (${\text{C}}_{{{\text{ds}}}}$) relative to a calibration step without biomolecules (${\text{C}}_{{{\text{ds}}\_0}}$), as shown in Eq. (18).

18 $$\begin{array}{*{20}c} {C {\text{response}} \left( {\text{\% }} \right) = 100\left( {\frac{{{\text{C}}_{{{\text{ds}}}} }}{{{\text{C}}_{{{\text{ds}}\_0}} }} - 1} \right)} \\ \end{array}$$

For N data (1,2,3,4,5, i,j,…..N) obtained in one test cycle, first, source-drain current of j state ($I_{ds\_j}$) minus that of i state ($I_{ds\_i}$) is the change of current between two states ($\partial I_{ds}$), as shown in Eq. (19).

19 $$\begin{array}{*{20}c} {I_{ds\_j} - I_{ds\_i} = \partial I_{{ds { }}} } \\ \end{array}$$

Then, $\partial I_{ds}$ divided by the change of gate voltage between two states ($\partial V_{g}$) is the corresponding slope value (${\text{S}}_{{\text{m}}}$), as shown in Eqs. (20a) and (20b).

20a $$\begin{array}{*{20}c} {V_{g\_j} - V_{g\_i} = \partial V_{g} } \\ \end{array}$$

20b $$\begin{array}{*{20}c} {{\text{S}}_{{\text{m}}} = \frac{{\partial I_{ds} }}{{\partial V_{g} }}} \\ \end{array}$$

Finally, the average of all slope values for a test cycle ($\overline{{{\text{S}}_{{\text{m}}} }}$) is taken to yield the ${\text{C}}_{{{\text{ds}}}}$ for that cycle, as shown in Eq. (21).

21 $$\begin{array}{*{20}c} {{\text{C}}_{{{\text{ds}}}} = \overline{{{\text{S}}_{{\text{m}}} }} } \\ \end{array}$$

The frequency used as indication signals has been counted from 2017 to 2022, as shown in Tables 1 and 2. We found that the Dirac voltage response is more widely chosen as the indication signal than the current response in graphene-based FET biosensing. Researchers prefer to select the current response as the indication signal in MX_2_-based FET biosensing compared to the threshold voltage response. The capacitance response as the indication signal was first proposed in 2021 but rarely was reported in either graphene-based or MX_2_-based FET biosensing. The capacitance response is promising to expand indication signal types.

## Optimization strategies of FET

With the development of nanotechnology and biotechnology, various strategies to optimize the biosensing performance of FET have been widely reported, but not been summarized in technical detail until now. In order to help researchers learn about the current optimization direction and master the current technological progress, the optimization strategies of sensing materials, probe immobilization methods, probe types, and introducing signal amplifying groups were reviewed and dialectically evaluated.

### The exploration in sensing material

We reviewed the strategy to improve the sensitivity of FET by optimizing the sensing material from three directions. Direction 1: exploring types of the sensing material; Direction 2: optimizing transfer processes of the sensing material; Direction 3: exploring configurations of the sensing material.

#### Exploring types of the sensing material

Graphene or MX_2_ selected as the sensing material of FET for biomolecular detection has been widely reported to optimize the sensing performance [111, 121].

**Fig. 3:**
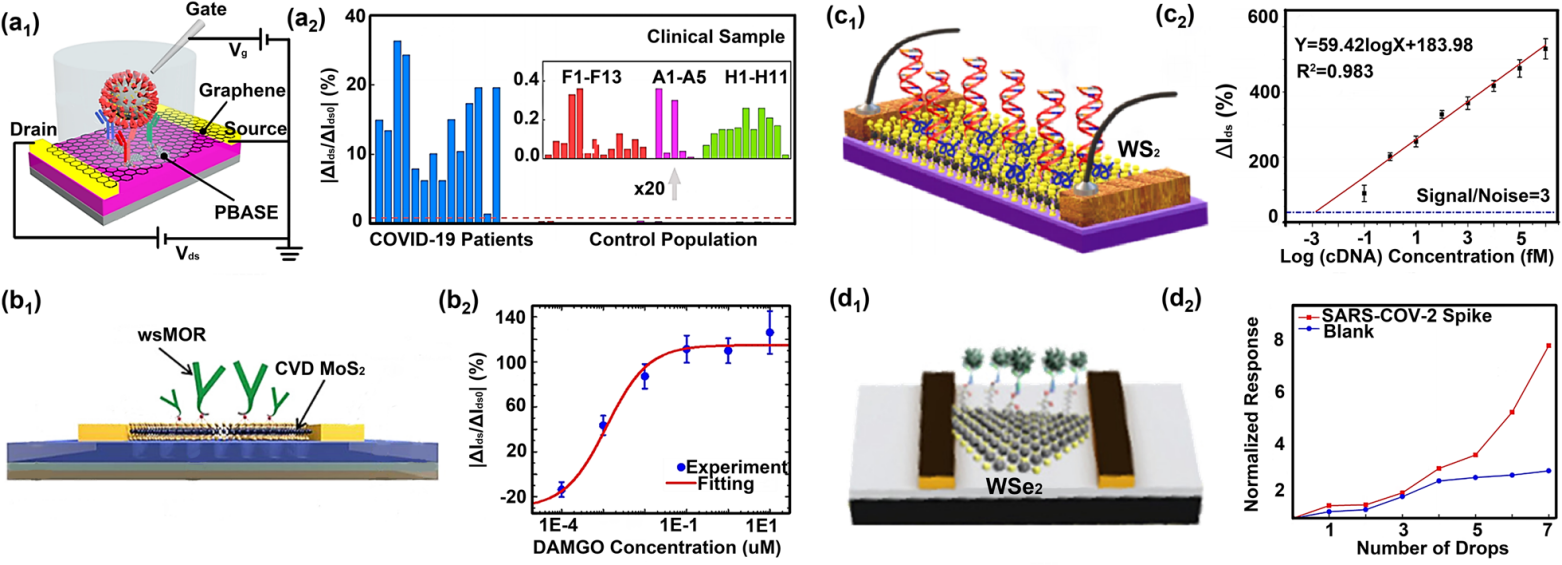
Optimization strategies from the direction of sensing material types. **a**_**1**_ The graphene-based FET detecting SARS-COV-2 characteristic protein. **a**_**2**_ Dynamic response of this biosensor to SARS-COV-2 characteristic protein almost 100%. **b**_**1**_ The MoS_2_-based FET detecting polypeptide receptor wsMOR. **b**_**2**_ The detection range of this biosensor to wsMOR from 0.1 nM to 10 uM. **c**_**1**_ The WS_2_-based FET detecting DNA hybridization. **c**_**2**_ The response range of this biosensor to target DNA from 10^−16^ M to 10^−9^ M. **d**_**1**_ The WSe_2_-based FET detecting SARS-COV-2 characteristic protein. **d**_**2**_ The detection range of this biosensor to SARS-COV-2 characteristic protein from 25 fg/ul to 10 ng/ul. **a** Reproduced with permission [88]. Copyright 2021, American Chemical Society. **b** Reproduced with permission [127]. Copyright 2019, Institute of Physics Science. **c** Reproduced with permission [105]. Copyright 2022, American Chemical Society. **d** Reproduced with permission [106]. Copyright 2021, American Chemical Society

The lattice structure of monolayer graphene has minimal hindrance to electron transport. The carrier mobility is as high as 2 × 10^5^ cm^2^V^−1^ s^−1^ [128], two orders more elevated than silicon, indicating the graphene-based FET has a high sensitivity to detect charged molecules [129]. Kobayashi’s team proposed a monolayer graphene-based FET to detect biotin [116]. In their work, this FET achieved a biotin detection sensitivity as low as 0.37 pM and readily distinguished target biomolecules from real samples, indicating high selectivity. Dai et al. proposed a monolayer graphene-based FET to detect SARS-COV-2 characteristic protein (Fig. 3a1) [88]. In their work, this FET achieved ultrasensitive SARS-CoV-2 spike antibody detection with a LOD of 0.34 fM, and detected clinical serum samples with a diagnostic sensitivity almost 100% (Fig. 3a2). Kumar et al. proposed a monolayer graphene-based FET to detect carbonic anhydrase 1 (CA1) [130]. This FET realized CA1 detection with a broad response range from 10 pg/ml to 100 ng/ml. With the development of nanotechnology, except for graphene, other 2D materials with high electrical response sensitivity have also been reported. Researchers attempted to apply these newly discovered 2D materials to the sensing material of FET for biosensing.

Compared to zero-bandgap graphene, the transition metal chalcogenides (MX_2_) is also an excellent choice to be used as sensing material of FET due to their adjustable bandgap and larger switching ratio [15, 131, 132]. Wei et al. proposed a multilayered MoS_2_-based FET to detect β-actin antibodies [122]. Here, this proposed FET realized β-actin antibody detection ranging from 10^−9^ to 10^−3^ μg/μL and showed a selective response toward multiple proteins. Eknamkul et al. proposed a monolayer MoS_2_-based FET to detect polypeptide receptor wsMOR (Fig. 3b1) [127]. In their work, this MoS_2_ FET realized wsMOR detection with a LOD of 1 nM and exhibited a broad detection range from 0.1 nM to 10 uM (Fig. 3b2). Bahri et al. proposed a monolayer WS_2_-based FET to detect DNA hybridization (Fig. 3c1) [133]. Here, the proposed FET realized ultrasensitive DNA hybridization detection with a LOD of 3 aM and a broad detection range from 10^−16^ to 10^−9^ M (Fig. 3c2) and showed a selective response toward one-base, two-base, and three-base mismatched DNA. Hafshejani et al. proposed a monolayer WSe_2_-based FET to detect SARS-COV-2 characteristic protein (Fig. 3d1) [106]. In their work, this FET realized ultrasensitive SARS-CoV-2 spike antibody detection with a LOD of 25 fg/uL and had a selective response toward BSA and SARS-CoV-2 antigen protein (Fig. 3d2). These FETs using different 2D materials as the sensing material exhibit good biosensing properties in aspects of sensitivity, specificity, and stability.

Researchers can choose the type of sensing material suitable for their experimental needs according to the sensing demands, the preparation process of sensing material, and the cost.

#### The emerging trends in the synthesis of 2D materials

Liang et al. reviewed that the optimal performance of FET devices relies heavily on the quality of the sensing material [134]. Zhang et al. also emphasized that monolayer 2D materials were shown to be particularly effective in improving the detection capabilities of FET biosensors due to their unique properties [135], such as higher carrier mobility and fewer defects compared to multilayer materials. Therefore, it is essential to synthesize high-quality 2D materials to achieve excellent detection capabilities in 2D materials-based FET biosensors. Various methods of synthesizing 2D materials have been reported with the development of material synthesis technology. Currently, the most widely used methods for obtaining 2D materials are mechanical exfoliation [136], liquid-phase exfoliation [137], and chemical vapor deposition [138].

Cheng et al. reviewed that mechanical exfoliation was a viable method of obtaining 2D materials by peeling off thin layers of the material [139]. While this technique was suitable for primary research purposes, it was expensive, complicated, and impractical for large-scale production. Witomska et al. reviewed that liquid-phase exfoliation was a cost-effective method for obtaining 2D materials, which used solvents to extract thin layers of material from bulk samples [140]. However, this technique had limitations in producing monolayer 2D materials and obtaining large-sized samples. Qin et al. reviewed that chemical vapor deposition (CVD) was an important method for synthesizing high-quality 2D materials [141]. This method involved introducing a precursor gas into a reactor chamber and heating it to form a thin material layer on a substrate. Deng et al. concluded the advantages of the CVD method to synthesizing 2D materials [142] because it allowed for controllable synthesis conditions, making it easy to produce high-quality monolayer materials and achieve large-scale production. As a result, it is an ideal choice for applications requiring high-quality sensing materials.

Choosing a suitable method for synthesizing 2D materials depends on the specific application requirements. For example, the CVD method may be the most suitable for large-scale production, while the mechanical exfoliation method may be preferred for producing high-quality monolayer materials for research purposes. Therefore, it is crucial to consider the specific needs of the application when selecting a method for synthesizing 2D materials. Further research and development are crucial to discovering new synthesis techniques and advancing 2D material-based FET biosensors. Through continuous effort, more advanced 2D material-based FET biosensors can be developed, leading to the discovery of new applications in the biosensing field.

#### Optimizing transfer steps of the sensing material

Recently, various methods of transferring sensing materials from the growth substrate to FET have been reported to optimize sensing performance.

**Fig. 4:**
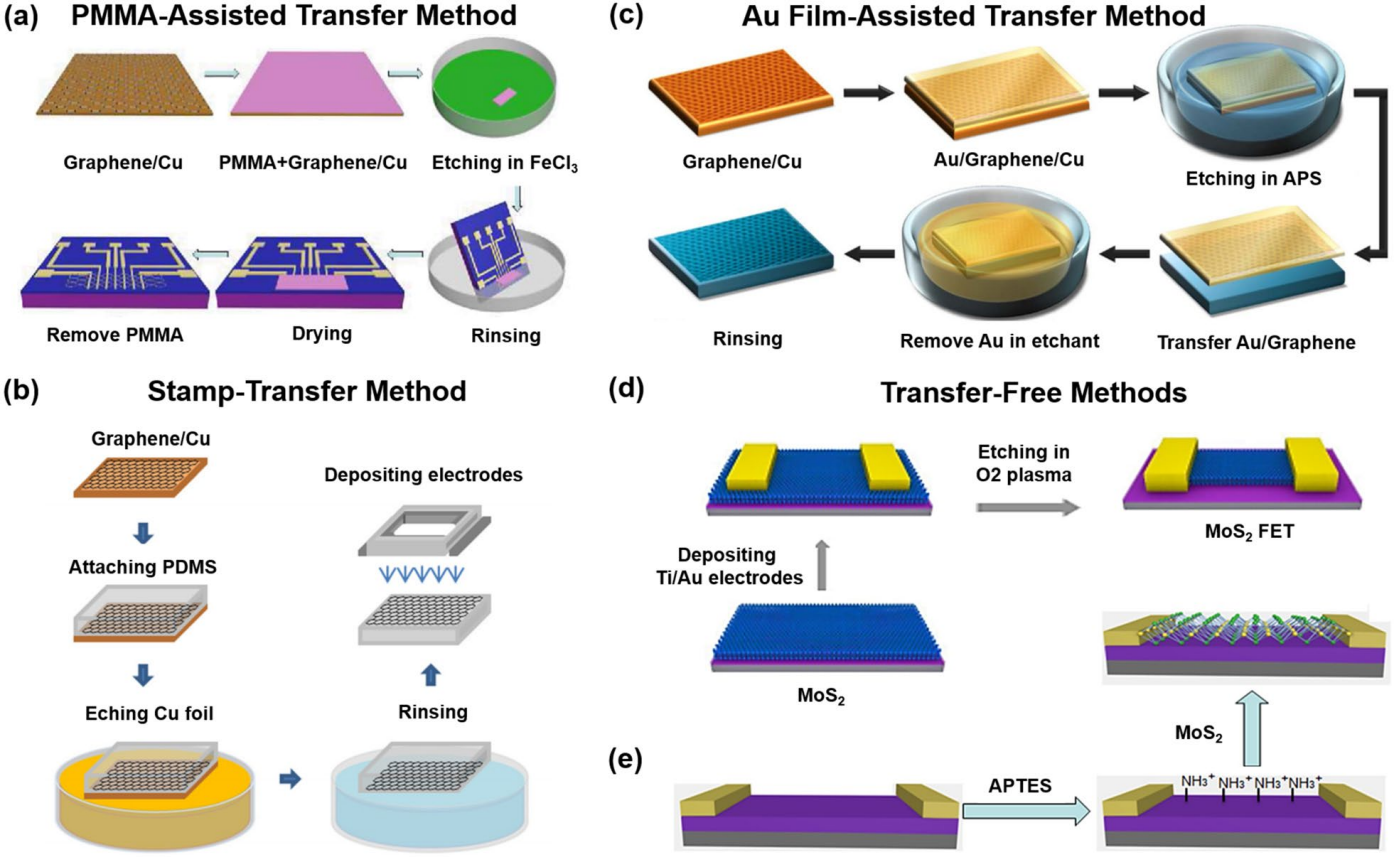
Optimization strategies from the direction of sensing material’s transfer steps. **a** PMMA-assisted transfer of graphene from the growth substrate to the target substrate. **b** Stamp-transfer graphene from the growth substrate to the target substrate. **c** Au film-assisted transfer of graphene from the growth substrate to the target substrate. **d** Growing MoS_2_ on the target substrate with transfer-free. **e** Depositing MoS_2_ on the APTES-modified target substrate with transfer-free. **a** Reproduced with permission [143]. Copyright 2015, American Chemical Society. **b** Reproduced with permission [144]. Copyright 2013, American Institute of Physics. **c** Reproduced with permission [145]. Copyright 2018, Elsevier Ltd. **d** Reproduced with permission [15]. Copyright 2019, American Chemical Society. **e** Reproduced with permission [16]. Copyright 2018, Elsevier Ltd

1. The naked eye-observed transfer method: it is the first reported to transfer graphene from the growth substrate. The graphene/copper substrate is firstly etched by FeCl_3_ solution. Researchers identified the graphene film floating on the surface of the cleaning solution (DI water) with the naked eye and transferred it to the target substrate. This naked eye-observed transfer method has a low cost of transferring graphene and does not dope graphene, but it takes a lot of time to find the graphene film in the cleaning solution due to the high light transmittance of graphene, and the graphene film is prone to cracks and wrinkles.
2. The PMMA-assisted transfer method (Fig. 4a): PMMA is widely used as the support layer to optimize the transfer steps of graphene [146, 147]. Firstly, the PMMA layer is coated on the graphene surface before transferring graphene from the growth substrate. After the growth substrate is etched, the PMMA + graphene is transferred to the target substrate together, and the PMMA film is removed by acetone treatment [143]. This method protects the graphene film from breaking during the transfer process and is convenient for researchers to quickly find the graphene film in the cleaning solution. But in this method, PMMA, cleaning solution, and acetone may cause chemical doping to the graphene film, reducing the electrical sensitivity of graphene.
3. The Ar plasma cleaning method: our team used an Ar plasma to remove PMMA absorbed on graphene film [84]. Here, the sensitivity of the Ar plasma-treated graphene FET (LOD of 1 aM) is approximately one order of magnitude higher than that of untreated graphene FET (LOD of 10 aM). This method can clean the graphene surface injury-free but is more complicated than the PMMA-assisted transfer method.
4. The stamp-transfer method (Fig. 4b) [144]: The target substrate is posted on the graphene surface before transferring graphene from the growth substrate. After the growth substrate is etched, the target substrate + graphene are acquired together. Compared with the PMMA-assisted transfer method, this method can avoid possible chemical contamination and decrease carrier mobility during graphene transfer.
5. The Au film-assisted transfer method (Fig. 4c) [145]: the gold film is evaporated on the graphene surface before transferring graphene from the growth substrate. After the growth substrate is etched, the gold film + graphene is transferred to the target substrate together, and the gold film is removed by the KI + Iodine solution. The Au-transferred graphene FET response signal is more significant (125%) than the PMMA-transferred graphene. Compared with the PMMA-assisted transfer method, this method makes the graphene surface clean and smooth, and avoids any potential chemical doping, but it has shortcomings such as high cost and complicated preparation process. Barreiro et al. used the current annealing method to remove contamination adsorbed on the graphene surface [148], further improving the carrier mobility of graphene. Here, the graphene surface with current annealing is smoother than without current annealing, but it cannot clean the PMMA residues thoroughly.

Transfer-free methods refer to techniques that allow for the direct growth of 2D materials on a target substrate or deposition of the material onto another layer without requiring the material to be transferred from its original substrate. The transfer-free method offers several benefits, including avoiding potential damage (such as wrinkle, fracture, et al.) to the sensing material, minimizing contamination and defect formation, and simplifying the overall fabrication process. For example, growing graphene directly on the target substrate can effectively avoid the defects and impurities of graphene during the transfer process. The sensitivity of this graphene FET is one order of magnitude higher than that of PMMA-transfer graphene FET [66]. Liu et al. used the chemical vapor deposition (CVD) method to grow MoS_2_ on the target substrate (Fig. 4d). Here, this transfer-free method maximizes the preservation of the performance of the grown sensing material [15]. Depositing MoS_2_ directly on the APTES-modified target substrate is a good idea to avoid transfer steps (Fig. 4e). Here, the authors pre-modified a layer of positive charges on the target substrate to enable an efficient binding between the target substrate and MoS_2_ via electrostatic interaction [16].

The above three transfer-free methods can effectively avoid the shortcoming of exogenous chemical doping, cracks, and wrinkles of the sensing material during the transfer process. Compared with the naked eye-observed transfer method, the PMMA-assisted transfer method, the Au-assisted transfer method, and the Ar plasma cleaning method, the transfer-free method is the best method to fabricate the sensing material of FET.

#### Exploring configurations of the sensing material

**Fig. 5:**
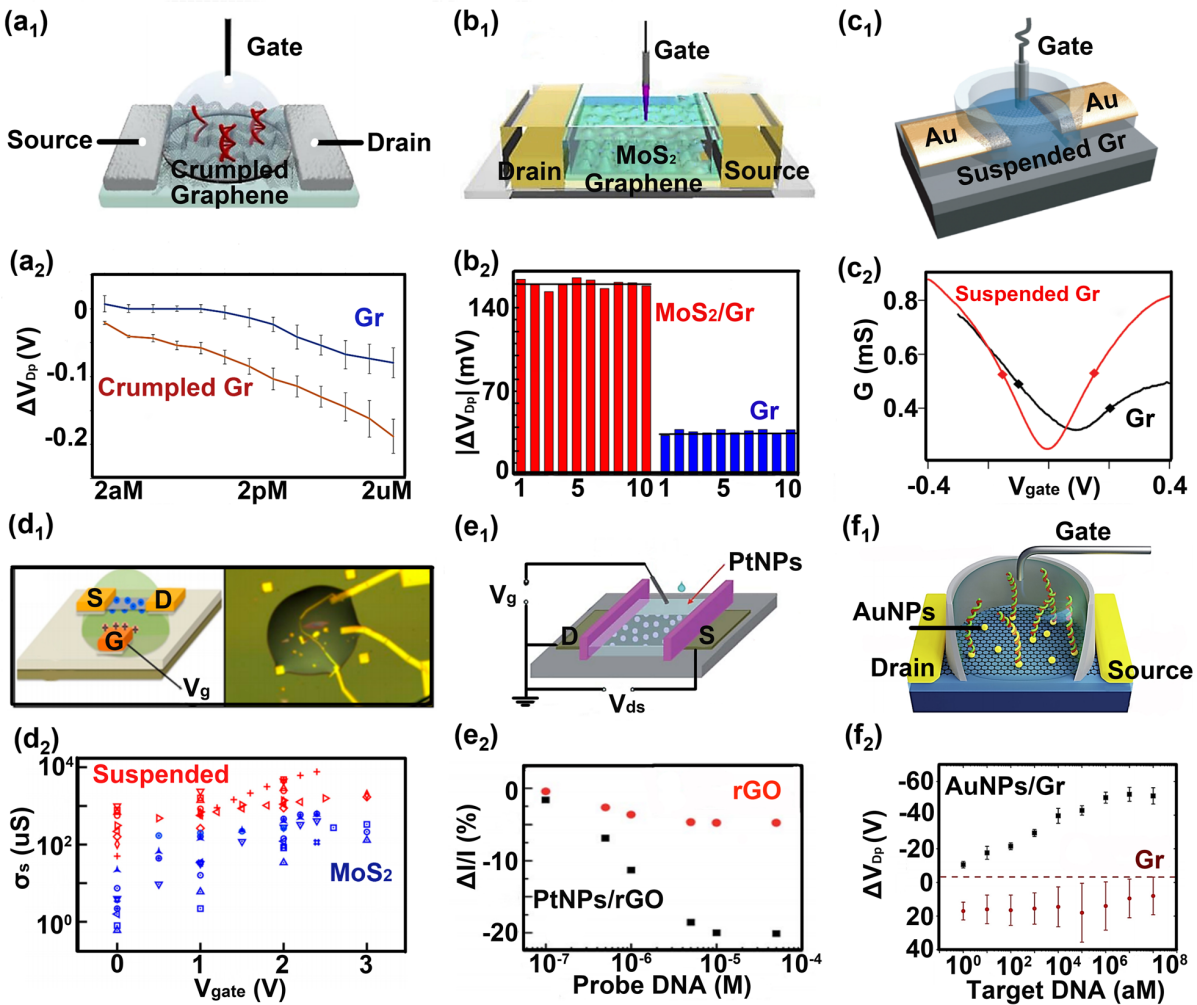
Optimization strategies from the direction of sensing material configurations. **a**_**1**_ The wrinkled graphene-based FET detecting DNA hybridization. **a**_**2**_ The detection range of this biosensor to target DNA from 2 aM to 2 uM. **b**_**1**_ The MoS_2_/graphene-based FET detecting DNA hybridization. **b**_**2**_ The response signal of this biosensor to probe DNA is four times that of graphene FET. **c**_**1**_ The suspended graphene-based FET detecting HF. **c**_**2**_ The carrier mobility of suspended graphene is two times that of graphene contacted with the substrate. **d**_**1**_ The suspended MoS_2_-based FET detecting charged ions. **d**_**2**_ The conductance of the suspended MoS_2_ is 1 − 2 orders of magnitude higher than that of the MoS_2_ supported with the substrate. **e**_**1**_ The platinum nanoparticles/reduced graphene oxide-based FET detecting DNA hybridization. **e**_**2**_ The response signal of this biosensor to probe DNA is five times that of graphene FET. **f**_**1**_ The gold nanoparticles/graphene-based FET detecting DNA hybridization. **f**_**2**_ The detection range of this biosensor to target DNA from 1 aM to 1 pM. **a** Reproduced with permission [4]. Copyright 2020, Nature Publishing Group. **b** Reproduced with permission [31]. Copyright 2020, Elsevier Ltd. **c** Reproduced with permission [149]. Copyright 2010, American Chemical Society. **d** Reproduced with permission [150]. Copyright 2015, American Chemical Society. **e** Reproduced with permission [151]. Copyright 2012, Royal Society of Chemistry. **f** Reproduced with permission [92]. Copyright 2020, Elsevier Ltd

With the development of nanotechnology, multiple configurations with different sensing advantages were selected as the sensing material to enhance the biosensing performance of FET from different directions. Hwang et al. proposed a wrinkled graphene-based FET to detect DNA hybridization (Fig. 5a1) [4]. In their work, graphene can be curved at the micrometer-scale and nanometer-scale to form crumpled graphene, increasing the Debye length and allowing more biomolecules to be detected [117], so the sensitivity of crumpled graphene FET (2 aM) is six orders of magnitude higher than that of flat graphene FET (2 pM) (Fig. 5a2). Similarly, Park et al. proposed a crumpled graphene-based FET to detect SARS-CoV-2 virus amplification [152]. Here, the deep, narrow trench on twisted graphene can provide low ionic screening for an absorbed DNA molecule with increasing EDL length. Li et al. proposed an MXene/graphene-based FET to detect SARS-COV-2 characteristic protein and influenza virus [77]. In their work, this FET fully combined the electrical response sensitivity of graphene with the high chemical sensitivity of MXene, so realized the ultrasensitive detection for the influenza virus with a concentration of 125 copies/mL and the recombinant 2019-nCoV spike protein with a concentration of 1 fg/mL. Our team proposed a MoS_2_/graphene-based FET to detect DNA hybridization (Fig. 5b1) [31]. Here, monolayer graphene is used as the sensing material, and MoS_2_ is used as a protective layer to reduce the noise signal caused by the disturbance of water molecules on graphene. Thus the response signal of MoS_2_/graphene FET is four times that of graphene FET (Fig. 5b2).

The sensing material of FET is usually in direct contact with the substrate, so the atmospheric gases, unknown functional groups, chemical adsorbates, and ripple charges absorbed on the substrate surface reduce the electrical response performance of the sensing material [153]. Although the substrate was carefully cleaned before using it, the cleaning process cannot completely eliminate the adverse effect of impurities on the sensing material. Suspending the sensing material without touching the substrate is a good idea to eliminate the adverse effect caused by impurities absorbed on the substrate. Cheng et al. proposed a suspended graphene-based FET to detect HF (Fig. 5c1) [149]. Here, the carrier mobility of suspended graphene is twice that of graphene contacted with the substrate (Fig. 5c2). Wang et al. proposed a suspended MoS_2_-based FET to detect charged ions (Fig. 5d1) [150]. Here, the conductance of the suspended MoS_2_ is two orders of magnitude higher than that of the MoS_2_ supported with the substrate (Fig. 5d2). Similarly, in Taiyu Jin’s work [154], the carrier mobility and switching ratio of the suspended MoS_2_ are 2 and 10 times higher than those of the MoS_2_ contacted with the substrate.

The structure of graphene or MoS_2_ complex gold or platinum nanoparticles effectively increases the sensing area and enhances the sensing material's conductivity. Yin et al. proposed platinum nanoparticles/reduced graphene oxide-based FET to detect DNA hybridization (Fig. 5e1) [151]. In their work, the probe density on nanoparticles/reduced graphene oxide surface is larger than the reduced graphene oxide surface due to a larger sensing area (Fig. 5e2). Liu et al. proposed a gold nanoparticles/MoS_2_-based FET to detect related DNA of Down syndrome [15]. This FET realized an ultrasensitive detection for the chromosome 21 or 13 DNA fragment with a LOD of 100 aM and showed a selective response toward three-base mismatched DNA. Danielson et al. proposed a gold nanoparticles/graphene-based FET for detecting DNA hybridization (Fig. 5f1) [92]. This FET presented a broad detecting range from 1 aM to 1 pM, effectively discriminating between a complementary strand and a single nucleotide polymorphism (SNP) containing strand (Fig. 5f2). Li et al. proposed a gold nanoparticles/graphene-based FET for detecting SARS-COV-2 RNA [99]. In their work, this FET presented a low limit of SARS-COV-2 RNA detection in PBS (0.37 fM), throat swabs (2.29 fM), and serum (3.99 fM).

### The exploration in probe immobilization methods

Probe immobilization methods with high stability refer to techniques that utilize high-stability polymers as the probe-fixed carrier to connect the probe to the sensing material through adsorption or chemical cross-linking. Such techniques enhance the reliability of the sensing system, making it more accurate. With the development of biochemical technology, varieties of probe immobilization methods were developed, and the stability of probes was continuously improved. Here, we summarize eight probe immobilization methods and point out their advantages and disadvantages for reference by researchers.

**Fig. 6:**
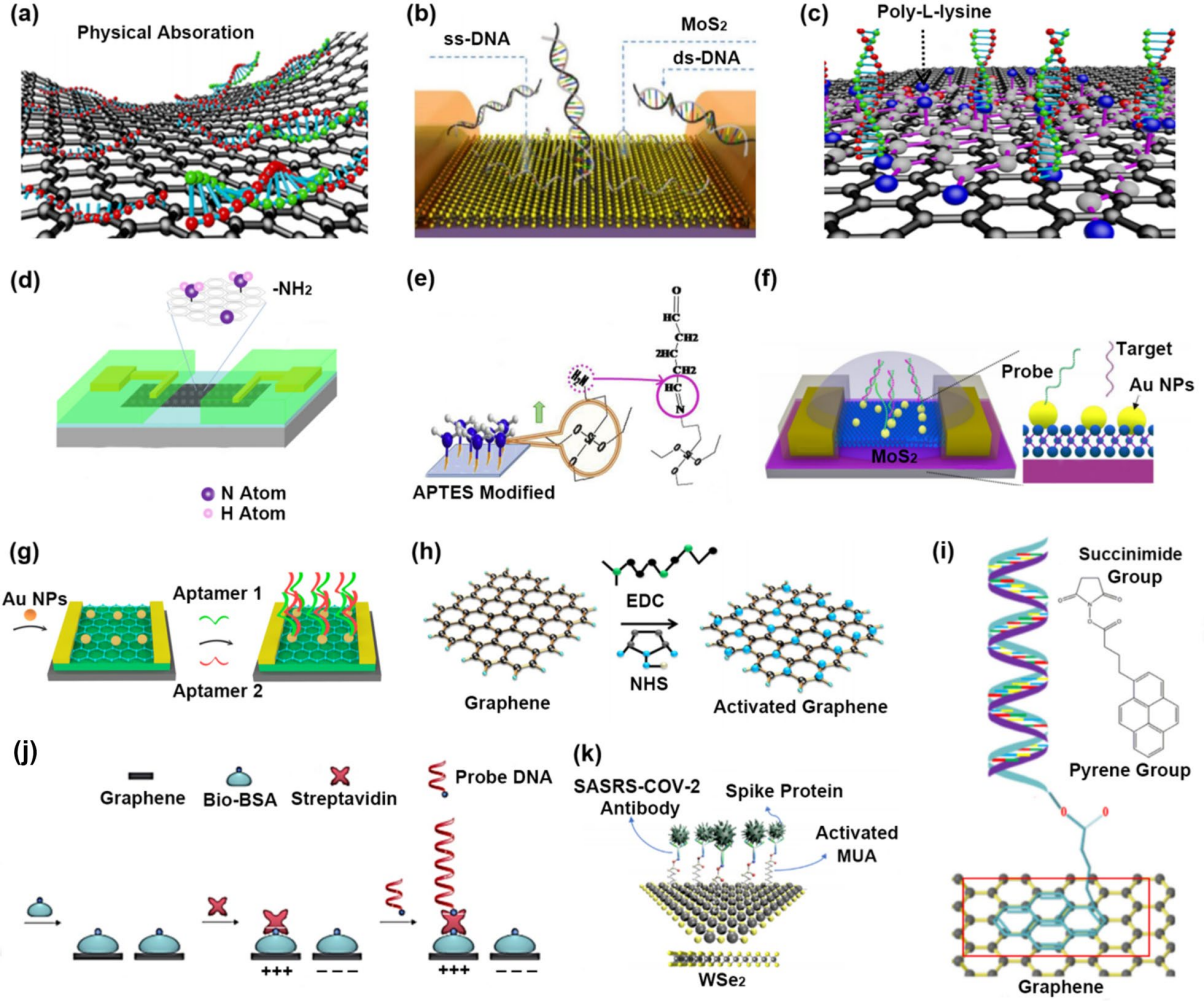
Optimization strategies from the direction of probe immobilization methods. **a**, **b** Immobilizing unmodified probes based on physical absorption. **c**, **d** Immobilizing unmodified probes based on electrostatic adsorption. **e** Immobilizing amino-modified probes based on glutaraldehyde cross-linking. **f**, **g** Immobilizing thiol-modified probes based on Au–S bond. **h** Immobilizing amino-modified probes based on EDC + NHS cross-linking. **i** Immobilizing amino-modified probes based on PBASE cross-linking. **j** Immobilizing biotin-modified probes based on Biotin-streptavidin cross-linking. **k** Immobilizing amino-modified probes based on MUA cross-linking. **a** Reproduced with permission [95]. Copyright 2020, American Chemical Society. **b** Reproduced with permission [101]. Copyright 2015, Royal Society of Chemistry. **c** Reproduced with permission [93]. Copyright 2022, American Chemical Society. **d** Reproduced with permission [94]. Copyright 2021, American Chemical Society. **e** Reproduced with permission [155]. Copyright 2018, Elsevier Ltd. **f** Reproduced with permission [15]. Copyright 2019, American Chemical Society. **g** Reproduced with permission [65]. Copyright 2020, American Chemical Society. **h** Reproduced with permission [119]. Copyright 2019, Elsevier Ltd. **i** Reproduced with permission [47]. Copyright 2021, Elsevier Ltd. **j** Reproduced with permission [156]. Copyright 2014, Nature Publishing Group. **k** Reproduced with permission [106]. Copyright 2021, American Chemical Society

1. The functionalization method of immobilizing probes based on physical adsorption [157, 158]: the single-stranded probe DNA was directly adsorbed on the surface of the sensing material through the π-π stacking between the base aromatic ring and the sensing material (Fig. 6a and b) [95, 101]. However, this method usually requires a long reaction time (~ 10 h) or extreme reaction conditions (-40 °C) to fix probes to the surface of the sensing material. Probes are easily desorbed from the surface of sensing material due to the low adsorption strength, resulting in unstable response signals. Moreover, this method can not avoid non-specific adsorption of target molecules to sensing material.
2. The functionalization method of immobilizing probes based on electrostatic adsorption [159]: the surface of the sensing material is modified with positively charged amino groups or other groups (such as PLL (Fig. 6c) [93]/NH^2+^ (Fig. 6d) [94]/APTES [77]) to increase the electrostatic attraction of the sensing material to the negatively charged probe. Compared with physical adsorption, this method enhances the adsorption strength of probes on the surface of the sensing material. However, some experimental results show that the probe is still desorbed from the surface of the sensing material, resulting in unstable response signals. Similar to physical adsorption, it's hard to determine whether the obtained signal is caused by the capture of the target molecule through the probe or the non-specific adsorption of the target molecule to sensing material.
3. The functionalization method of immobilizing probes based on glutaraldehyde cross-linking [160, 161]: one end of APTES is non -covalently connected to the graphene surface, and the amino group at the other end of APTES is connected to the aldehyde group of glutaraldehyde through the aldimine condensation (Fig. 6e) [155]. Another aldehyde group of glutaraldehyde is also connected with amino-modified probes by the aldimine condensation. This method avoids non-specific adsorption of target molecules and ensures the purity of the response signal.
4. The functionalization method of immobilizing probes based on Au–S bond or Pt–S bond [162–164]: metal nanoparticles (such as AuNPs (Fig. 6f) [15], PtNPs [151]) are first deposited on the surface of the sensing material. Then thiol-modified probes are immobilized on the surface of sensing material by Au–S (Fig. 6g) [104] or Pt–S covalent bond. However, this method's shortcoming is that nanoparticles do not completely cover the surface of the sensing material, so single-stranded target nucleic acid molecules are absorbed on the exposed sensing material through π-π stacking. But there are also methods (such as tween 20 [165, 166], PEG [167, 168]) to encapsulate the exposed sensing material to overcome the nonspecific adsorption problem.
5. The functionalization method of immobilizing probes based on Carbodiimide (EDC) + N-hydroxysuccinimide ester (NHS) cross-linking [169, 170]: Method 1: EDC first reacts with carboxyl groups on the surface of carboxylated graphene to form unstable EDC/carboxyl active intermediate esters, and then NHS reacts with EDC/carboxyl active intermediate esters to form stable NHS/carboxyl active intermediate esters. The NHS/carboxy active intermediate ester on the graphene surface reacts with the amino group of probes to immobilize probes by the aldimine condensation (Fig. 6h) [119]. Method 2: EDC + NHS first reacts with the phosphate group of probes to form a stable NHS/phosphate group active intermediate ester, which reacts with the amino group of aminated sensing material surface to immobilize the probes [171].
6. The functionalization method of immobilizing probes based on 1-Pyrenebutyric acid N-hydroxysuccinimide ester (PBASE) cross-linking [172, 173]: the pyrene group of PBASE is linked to the graphene or MX_2_ surface through the π-π stacking, and the active NHS ester of PBASE is linked to the amino-modified probes through the amide bond (Fig. 6i) [47]. This method is the most widely selected to functionalize the sensing material of FET. But single-stranded target nucleic acid molecules are adsorbed on the pyrene group of PBASE through π-π stacking, resulting in nonspecific signals.
7. The functionalization method of immobilizing probes based on biotin-streptavidin cross-linking [174, 175]: the negatively charged biotin is non-covalently absorbed on the graphene surface, making the graphene surface covered by the negatively charged molecular layer. The positively charged streptavidin is then linked with biotin through the ELISA principle. Finally, the biotin-modified probes are linked to streptavidin (Fig. 6j) [156]. This method has two advantages: the positively charged biotin-streptavidin molecular layer formed on the graphene surface contributes to immobilizing negatively charged single-stranded probes (Advantage 1). It avoids the non-specific adsorption of target single-stranded nucleic acid molecules (Advantage 2).
8. The functionalization method of immobilizing probes based on 11-Mercaptoundecanoic acid (MUA) cross-linking [176, 177]: the sulfhydryl group at one end of MUA occupies the X vacancy of MX_2_. It is covalently linked to the MX_2_ surface. The carboxyl group of MUA reacts with the amino-modified probe by the amide bond (Fig. 6k) [106]. The X vacancy of MX_2_ is filled with the thiol groups of MUA, decreasing the defect state on the MX_2_ surface, but the higher the quality of MX_2_ material, the fewer X vacancies of the MX_2_ surface, indicating it is difficult for this method to achieve a good balance between the material performance and the probe density.

### The exploration in probe types

With the progress of biotechnology, various probe types were studied to optimize the sensing performance. Here, we summarize six probe types and point out the advantages and disadvantages of each method for researchers to refer to.

**Fig. 7:**
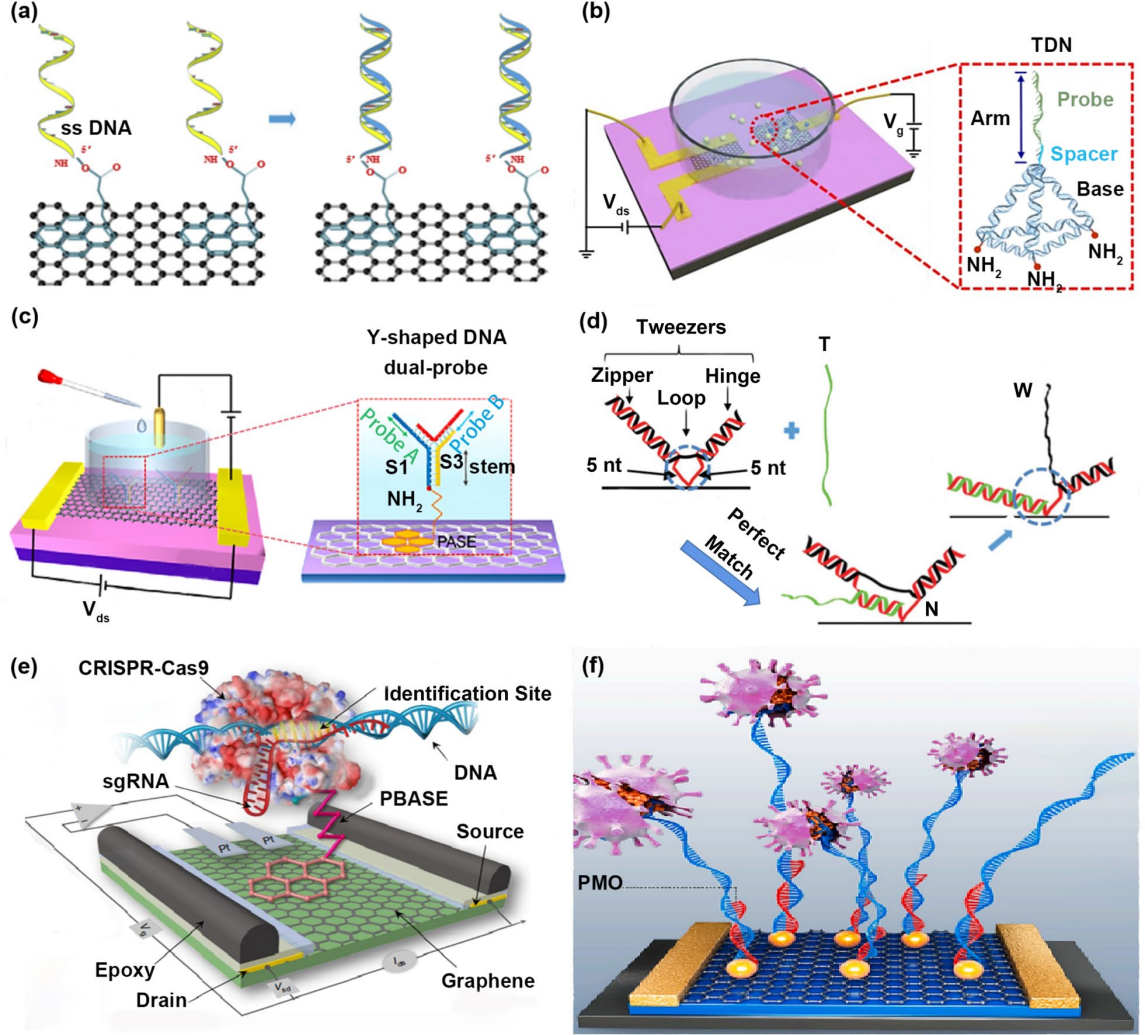
Optimization strategies from the direction of probe types. **a** Single-stranded nucleic acid probes. **b** Tetrahedral nucleic acid probes. **c** Y-shaped nucleic acid probes. **d** Hairpin-shaped nucleic acid probes. **e** Nucleic acid-protein composite probes. **f** Electrically neutral PMO probes. **a** Reproduced with permission [37]. Copyright 2019, Elsevier Ltd. **b** Reproduced with permission [50]. Copyright 2021, American Chemical Society. **c** Reproduced with permission [87]. Copyright 2021, American Chemical Society. **d** Reproduced with permission [79]. Copyright 2018, Wiley–VCH Verlag GmbH & Co. KGaA, Weinheim. **e** Reproduced with permission [3]. Copyright 2019, Nature Publishing Group. **f** Reproduced with permission [99]. Copyright 2021, Elsevier Ltd

1. Single-stranded nucleic acid probes [178, 179]: a single-stranded nucleic acid probe with a simple structure is a widely reported probe type (Fig. 7a) [37]. However, the local entanglement between single-stranded nucleic acid probes and the easy non-specific adsorption to the surface of the sensing material make a part of the probes inactivate, resulting in the low recognition efficiency of target molecules. Li’s team proposed a hall effect-based measurement method to prove the existence of the above shortcomings [180]. In their work, when single-stranded nucleic acid probes are used to identify target molecules, only a part of the base is paired with the base of targets. Other bases are adsorbed on the graphene surface instead of pairing with the base of targets.
2. Tetrahedral nucleic acid probes [111, 181]: the rigid base of the tetrahedral nucleic acid probe makes the single-stranded nucleic acid probe stand on the surface of the sensing material (Fig. 7b) [50]. In the tetrahedral structure, the single-stranded nucleic acid probes are spaced apart to avoid local entanglement between the probes. The rigid base of tetrahedron avoids non-covalent adsorption between single-stranded nucleic acid probes and the sensing material surface.
3. Y-shaped nucleic acid probes [182, 183]: the base of this Y-shaped probe is a rigid double-stranded DNA, allowing the probe DNA to stand on the surface of the sensing material, which avoids the local entanglement between probes and decreases non-specific adsorption between probes and the sensing material. The head of this Y-shaped probe has two single-stranded DNA probes, improving target molecule recognition efficiency (Fig. 7c) [87].
4. Hairpin-shaped nucleic acid probes [184]: the combination of the Weak strand (W) and the Normal strand (N) forms a hairpin structure, including a zipper area, loop area, and hinge area. The Normal strand of the zipper and hinge area combines with the Weak strand to form a double-stranded structure. The Target strand (T) combines with the Normal strand of the zipper area and loop area to form a double-stranded structure, causing a strand displacement reaction between the zipper region and the loop region (Fig. 7d) [79]. Based on this strand displacement reaction, the single-base mismatch was successfully detected.
5. Nucleic acid-protein composite probes: this CRISPR-Cas9 composite probe consists of a Cas9 protein and a piece of single-stranded RNA. Here, the amino-modified Cas9 protein immobilizes the CRISPR RNA sequence by specifically recognizing targets on the surface of the sensing material (Fig. 7e) [3]. This CRISPR-Cas9 composite probe recognizes double-stranded target nucleic acids, filling the gap that traditional nucleic acid probes only identify single-stranded targets.

Compared with traditional single-stranded nucleic acid probes, structural DNA probes, such as tetrahedral DNA, Y-shaped DNA, hairpin-shaped DNA probes, and nucleic acid-protein composites, such as RNA-Cas 9 composite probes are called unique structure probes in FET biosensing. In contrast to single-stranded nucleic acid probes, these probes possess distinct structural features that allow them to capture targets more effectively.

6. Electrically neutral PNA/PMO probes [97, 185]: both the nucleic acid probe molecule and the target nucleic acid molecule are negatively charged, so the target nucleic acid molecule is subject to electrostatic repulsion when it is recognized by the nucleic acid probe molecule, resulting in low recognition efficiency. To eliminate electrostatic repulsion, electrically neutral peptide nucleic acids (PNA) and electrically neutral morpholine antisense oligonucleotides (PMO) [16] were widely reported as probe molecules to replace traditionally charged probes. Compared with traditional negatively charged nucleic acid probes, no-charged DNA analogs that remove the negatively charged phosphate groups but maintain the base structures are called electrically neutral probes, such as PNA and PMO probes, which are effective in capturing targets and minimizing electrostatic interactions between the probes and the sensing material or targets. PNA is a DNA/RNA analog where the pentose phosphodiester backbone of DNA is replaced by a neutral peptide chain amide 2-aminoethyl glycine bond, and the rest of PNA is the same as DNA. Since PNA is electrically neutral, there is no electrostatic repulsion between the probe molecule and the target molecule, so the recognition efficiency is greatly improved. In addition, the PNA-DNA/RNA hybridization is not affected by the salt concentration of the reaction system. However, the length of PNA probes is usually not more than 18 bases. The PNA probes rich in purines (A, G) have poor water solubility and are easy to self-polymerize, limiting the application of PNA probes to a certain degree. Similar to PNA probes, electrically neutral PMO probes are also used to replace negatively charged nucleic acid probes (Fig. 7f) [99]. The five-carbon sugar and phosphate groups of traditional nucleic acid probes are replaced by the methylene morpholino and phosphoramide groups of PMO, making PMO probes electrically neutral. Compared with PNA probes and traditional nucleic acid probes, the PMO has a higher solubility and a higher enzyme resistance in a liquid environment, and is more flexible in the sequence length and the type of bases. So the PMO probe has great potential to promote the progress of FET in biosensing.

We believe exploring a probe combining PMO with a Y-shaped or tetrahedral structure is highly promising to eliminate electrostatic repulsion, improve recognition efficiency, and avoid mutual entanglement between probes.

### The exploration in multiplying target signals

**Fig. 8:**
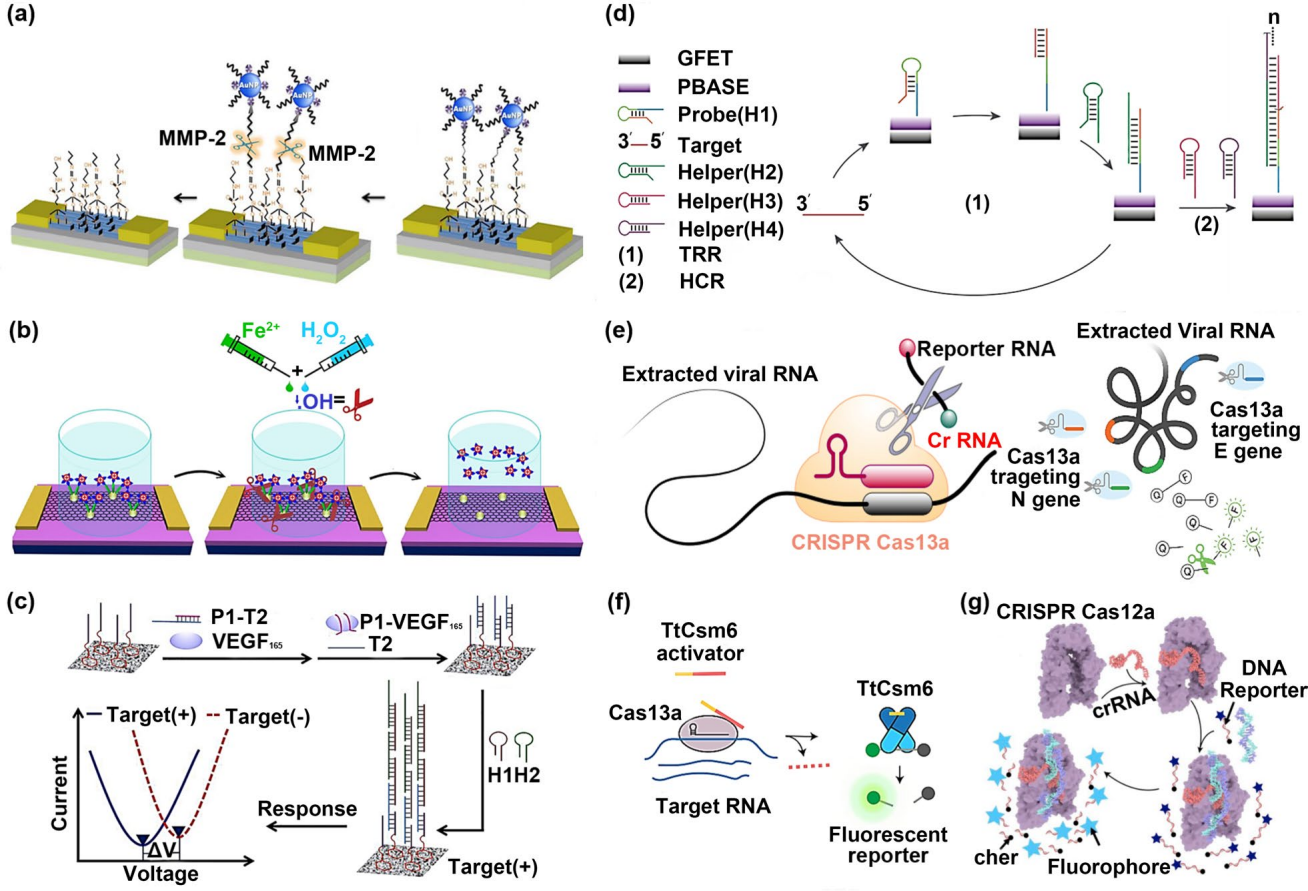
Optimization strategies from the direction of multiplying target signals. **a** Multiplying target signals based on the MMP-2-cutting reaction between the probe peptide sequence and MMP-2; **b** based on the·OH-cutting reaction between the cysteamine and·OH; **c** based on the VEGF165-catalyzed HCA; **d** based on the target DNA-catalyzed HCA; **e** based on the CRISPR-Cas13a system; **f** based on the CRISPR-Cas13a/Csm6 synergistic system; **g** based on the CRISPR-Cas12a system. **a** Reproduced with permission [186]. Copyright 2013, American Chemical Society. **b** Reproduced with permission [187]. Copyright 2019, Nature Publishing Group. **c** Reproduced with permission [44]. Copyright 2022, Elsevier Ltd. **d** Reproduced with permission [68]. Copyright 2018, American Chemical Society. **e** Reproduced with permission [188]. Copyright 2021, Elsevier Ltd. **f** Reproduced with permission [189]. Copyright 2021, Nature Publishing Group. **g** Reproduced with permission [190]. Copyright 2020, Elsevier Ltd

In addition to optimizing sensing materials, functionalization methods, probe types, etc., various strategies for multiplying target signals were explored to improve the response performance of FET biosensing. The essence of response signals dominated by the hybridization-driven target molecule grabbing mechanism is the signal accumulation, not the signal multiplication. Oh et al. proposed a signal multiplication strategy based on the MMP-2-cutting mechanism instead of a grabbing mechanism to amplify the response signal of MMP-2 (Fig. 8a) [186]. In this strategy, the target molecule is not captured by the probe molecule but cuts the signal group of probes that has a stronger signal generation capability from the surface of the sensing material, realizing the amplification of the response signal. Here, the signal group (DNA-gold nanoparticle complex) was immobilized on the surface of the sensing material by peptide chain cross-linking. The target MMP-2 degrades the peptide chain, resulting in the disappearance of signal groups from the surface of the sensing material. Wang et al. proposed a signal multiplication strategy based on the ·OH-cutting mechanism to amplify the response signal of the target hydroxyl radical (·OH) [187]. Their work immobilized the signal group (metal ions) on the graphene surface through cysteamine cross-linking (Fig. 8b). The cysteamine was degraded by the target hydroxyl radical, causing the disappearance of metal ions as signal groups from the graphene surface. Liu et al. proposed a signal multiplication strategy based on the BPA-cleavage mechanism to amplify the response signal of BPA molecules [191]. Here, the Au–S bond immobilizes the signal group (double-stranded DNA) on the graphene surface. Then the target BPA molecule breaks the Au–S bond, resulting in detaching of signal groups from the graphene surface.

Hybridization chain reaction (HCR) can amplify target signals and is widely used in FET biosensing. Chen et al. proposed a signal multiplication strategy based on a VEGF165-catalyzed hybridization chain reaction to amplify the response signal of target VEGF165 molecules (Fig. 8c) [44]. In their work, target molecules make the secondary DNA strand released from the double-stranded DNA-aptamer complex owing to the higher affinity of the aptamer to VEGF165, triggering the hybridization chain reaction to growing capture exogenous DNA groups for signal amplification. Similarly, Gao et al. proposed a signal multiplication strategy based on a target DNA-catalyzed HCA to amplify the response signal of target DNA molecules (Fig. 8d) [68]. In Lizhen Chen's work [44], one target molecule only triggers the hybridization chain reaction of one probe strand, thereby amplifying the response signal of target molecules. However, the target molecule of Zhaoli Gao's work is recycled [68]. One target molecule catalyzes the hybridization chain reaction of multiple probe strands, further amplifying the response signal of target molecules.

Gene-editing technologies based on CRISPR-Cas12 and CRISPR-Cas13 systems have shown great advantages in amplifying target signals and have attracted widespread attention due to their high specificity and superior degradation ability to report probes [192]. Fozouni et al. proposed a CRISPR-Cas13a system to detect SARS-COV-2 RNA (Fig. 8e) [188]. In their work, this CRISPR-Cas13a system consists of a Cas13a protein and a single-stranded RNA fragment. The CRISPR RNA sequence recognizes the target ssRNA, activating the cleavage ability of the Cas13a protein to single-stranded RNA. A single activated Cas13a protein cleaves the surrounding fluorescent reporter probes in arbitrarily large amounts, amplifying the response signal of the target ssRNA. Doudna’s team proposed a CRISPR-Cas 13a/Csm6 synergistic system to detect SARS-COV-2 RNA (Fig. 8f) [189]. Here, the synergistic system consists of a CRISPR-Cas13a system and a Csm6 endonuclease. The Csm6 endonuclease of this synergistic system has two advantages to amplifying the response signal of the target ssRNA: Advantage 1: Csm6 endonuclease further activates the cleavage ability of activated Cas13a protein. Advantage 2: The Csm6 endonuclease cleaves the surrounding fluorescent reporter probe in large quantities with the activated Cas 13a protein. He et al. proposed a CRISPR-Cas 12a system to detect African swine fever virus (ASFV) dsDNA (Fig. 8g) [190]. In their work, this CRISPR-Cas12a system consists of a Cas 12a protein and a single-stranded RNA fragment. The CRISPR RNA sequence recognizes the target dsDNA, activating the cleavage ability of the Cas12a protein to single-stranded DNA. A single activated Cas12a protein cleaves an arbitrarily large number of surrounding fluorescent reporter probes, thereby amplifying the response signal of the target dsDNA.

At present, the strategy of combining the CRISPR-Cas system with the FET system for biomolecular detection was not reported. The CRISPR-Cas system is promising to promote a breakthrough in the detection sensitivity of the FET system.

## Iterative strategies of FET

In recent years, various iterative strategies for promoting the integration and intelligent development of FET have been proposed. In order to help researchers better understand the current iterative strategies, we summarize the existing work from the combination of three directions with FET. Direction 1: microfluidic technology; Direction 2: microelectronics technology; Direction 3: wearable technology.

### Exploring the combination of microfluidics, microelectronics and FET

**Fig. 9:**
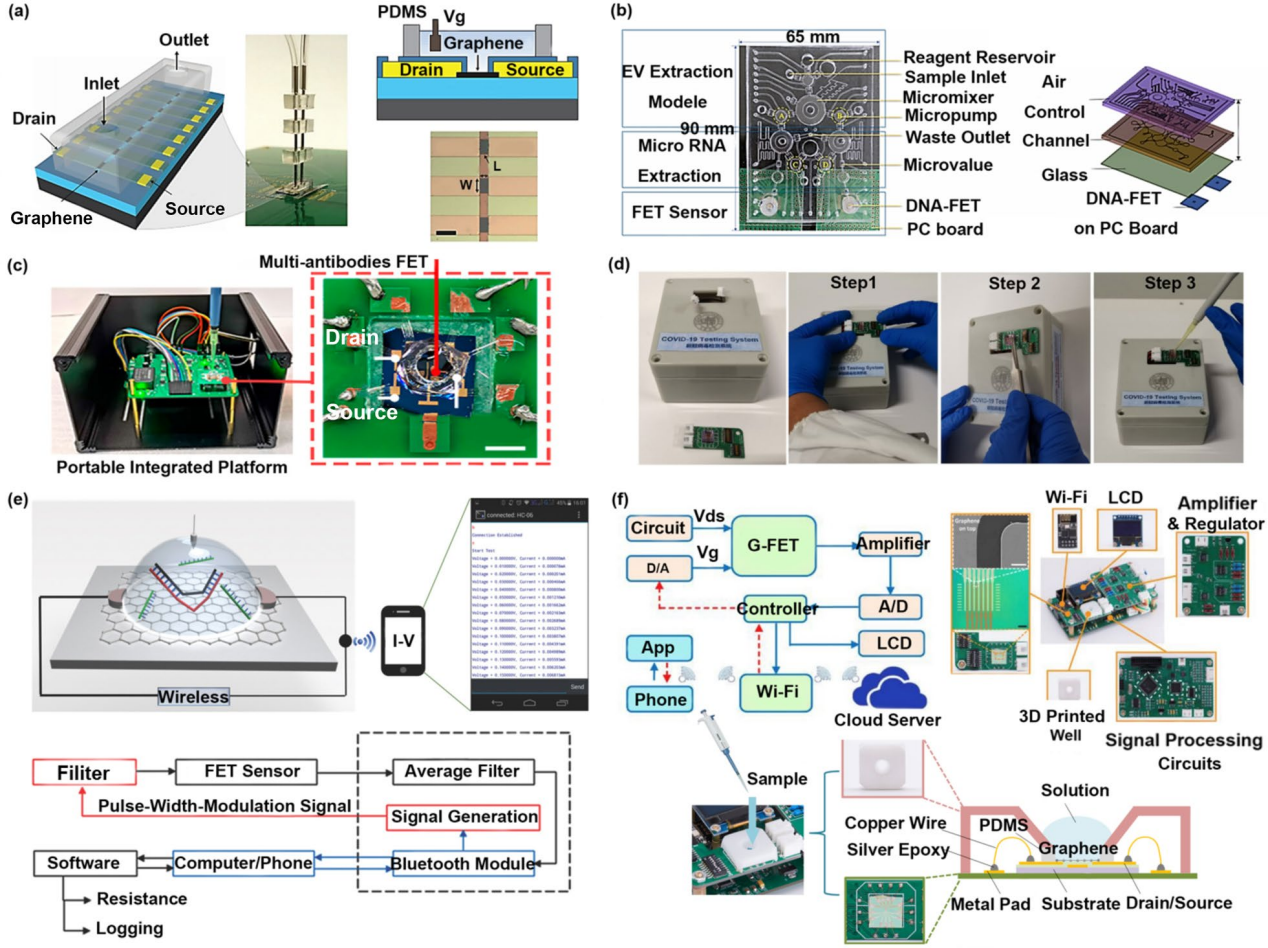
Iterative strategies for FET development using microfluidics and microelectronics. **a** Single-channel microfluidic chip for quantitative DNA hybridization detection. **b** Multifunctional microfluidic chip for miRNA detection. **c** Portable integrated platform for COVID-19 antigen detection. **d** Smart sensing platform for SARS-COV-2 RNA detection. **e** Intelligent analysis platform for single-base mismatch detection in DNA. **f** Intelligent sensing platform for cytokine biomarker detection in saliva. **a** Reproduced with permission [156]. Copyright 2014, Nature Publishing Group. **b** Reproduced with permission [193]. Copyright 2021, Springer-Verlag GmbH Germany. **c** Reproduced with permission [88]. Copyright 2021, American Chemical Society. **d** Reproduced with permission [1]. Copyright 2022, Nature Publishing Group. **e** Reproduced with permission [79]. Copyright 2018, Wiley–VCH Verlag GmbH & Co. KGaA, Weinheim. **f** Reproduced with permission [83]. Copyright 2019, Elsevier Ltd

Many strategies for the integration, intelligence, and on-site detection of FET enabled by microfluidics or microelectronics were gradually reported and effectively promoted the iterative development of FET [86, 194]. Ham’s team proposed a single-channel quantitative injection-based microfluidic chip to detect DNA hybridization [156]. Here, this microfluidic chip integrates a microfluidic channel and eight graphene FETs for multiplexed analysis (Fig. 9a). However, this microfluidic chip has a low integration and still relies on other equipment to separate and purify the sample. Kim et al. proposed a graphene-based portable device to detect gram-positive and gram-negative bacteria [195]. This device consists of a microfluidic chip, a microcontroller, a power supply, a communication module for outgoing data, an electronic circuit, and a portable rechargeable battery that can be used for the on-site monitoring of bacteria. Lee’s team proposed a microfluidic chip with the size of 65 mm*90 mm*5.7 mm to detect miRNA [193]. In their work, this microfluidic chip integrates a sample separation module, a purification module, and a sensing module (Fig. 9b), so the chip can not only realize quantitative injection but also be used for the separation and purification of mixed samples. Dai et al. proposed a portable integrated platform to detect the COVID-19 antigen (Fig. 9c) [88]. The platform integrates a testing module based on graphene FET, signal processing, and signal transduction circuits. It eliminates the limitations of large instruments and greatly meets the demand of on-site detection, promoting the portability and miniaturization of FET. Hajian et al. proposed a handheld-testing device to detect target single-nucleotide mutations relevant to two human disease models: SCD and ALS. This device can discriminate samples in 40 min through real-time, multi-parameter, and digital data acquisition in their work. Wang et al. proposed a smart sensing platform to detect SARS-COV-2 RNA, adenosine 5’-triphosphate, and thrombin (Fig. 9d) [1]. In their work, the platform integrates the MolEMS gFET-integrated testing module and the multifunctional system with a size of 11.5 cm * 9 cm * 5.5 cm, and is connected to a smartphone or computer via USB, WiFi, or Bluetooth, promoting the integration and intelligence of FET. Hwang et al. proposed an intelligent analysis platform based on graphene FET to identify single-base mismatches of DNA (Fig. 9e) [79]. In their work, the analysis platform consists of the user's electronic equipment and an intelligent detection platform with signal identification and Bluetooth wireless transmission functions. Michael T Hwang's work promotes the progress in online testing platforms of FET, shortens the distance between patients and doctors, and allows patients to have a more comfortable detection environment. Hao et al. proposed an intelligent sensing platform to detect cytokine biomarkers of saliva (Fig. 9f) [83]. In their work, the platform consisting of a sensing module and online signal processing circuits wirelessly transmits data information to a smartphone or cloud server via an internal Wi-Fi module, allowing doctors to remotely monitor patients, greatly promoting the intelligence of FET.

It is foreseeable that the portable smart-sensing device combining microfluidic technology and microelectronics technology with FET has excellent potential in on-site detection and is expected to promote the iterative development of FET.

### Exploring the combination of wearable technology and FET

**Fig. 10:**
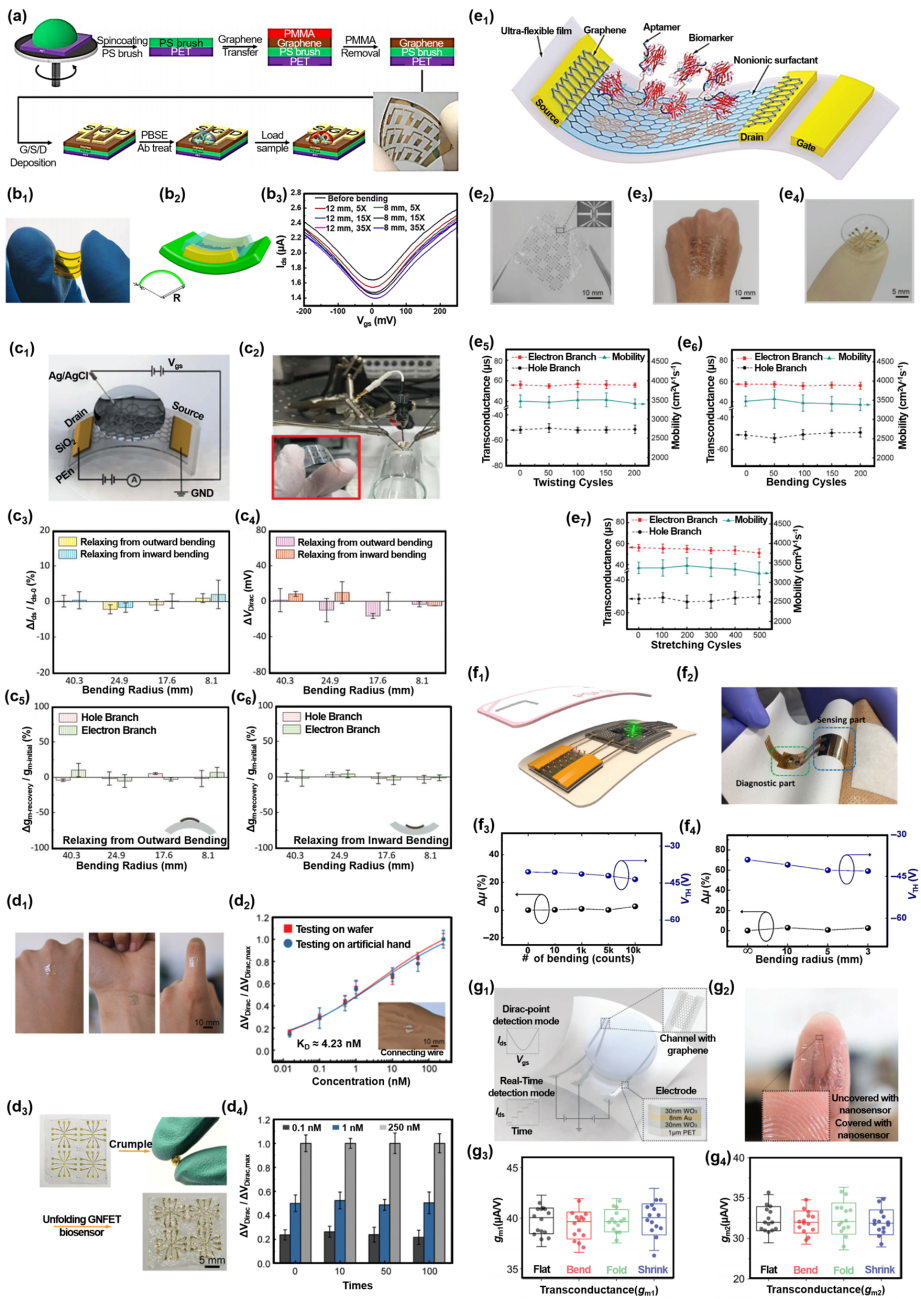
Iterative strategies of FET from the direction of wearable technology. **a** The flexible biosensor detecting the HIV-1 virus and MLV virus. **b** The wearable biosensor detecting related miRNA of breast cancer. **c** The flexible wearable sensor attached to the skin surface detecting the TNF-α of sweat. **d** The flexible wearable sensor attached to the wrist or finger surface detecting the IFN-γ of sweat. **e** The flexible wearable sensor attached to human tissue or skin surface detecting the TNF-α of sweat. **f** The epidermal skin-type point-of-care device detected PSA protein. **g** The wearable biosensor mounted on the eyeball detecting the L-cysteine of tears. **a** Reproduced with permission [90]. Copyright 2019, Institute of Physics Science. **b** Reproduced with permission [95]. Copyright 2020, American Chemical Society. **c** Reproduced with permission [80]. Copyright 2018, Royal Society of Chemistry. **d** Reproduced with permission [96]. Copyright 2021, Wiley–VCH Verlag GmbH & Co. KGaA, Weinheim. **e** Reproduced with permission [81]. Copyright 2019, Wiley–VCH Verlag GmbH & Co. KGaA, Weinheim. **f** Reproduced with permission [196]. Copyright 2017, Tsinghua University Press and Springer-Verlag Berlin Heidelberg. **g** Reproduced with permission [197]. Copyright 2022, Wiley–VCH GmbH

Iterative strategies combining FET with wearable technology were gradually reported, and more application scenarios of FET were explored [80, 198]. Kim et al. proposed a flexible biosensor to detect HIV-1 and MLV viruses (Fig. 10a) [90]. This wearable biosensor uses polyethylene terephthalate (PET) as a flexible substrate instead of the traditional rigid silicon substrate. Gao et al. proposed a wearable biosensor to detect related miRNA of breast cancer (Fig. 10b1 and b2) [95]. In their work, this wearable sensor uses polyimide (PI) as a flexible substrate, and the sensing performance is hardly changed after 35 bending cycles (bending radius of 8 mm; the tensile strain of 0.62%) (Fig. 10b3). Hao et al. proposed a flexible wearable sensor attached to the skin surface to detect the TNF-α of sweat (Fig. 10c1 and c2) [80]. Here, the wearable sensor uses polyethylene naphthalate (PEN) as a flexible substrate, and the sensing performance measured while bent (8.1 mm bending radius; 0.8% tensile and compressive strain) is the same as measured while flat (Fig. 10c3–c6), ensuring detection performance during human movement. Lee et al. proposed a stretchable sensor attached to human skin to detect the glucose in sweat [199]. Here, the stretchable device features a serpentine bilayer of gold mesh and gold-doped graphene that forms an efficient electrochemical interface for the stable transfer of electrical signals. Wang et al. proposed a flexible wearable sensor attached to the wrist or finger surface to detect the IFN-γ (Fig. 10d1 and d2) [96]. In their work, the wearable sensor uses polyethylene terephthalate as a flexible substrate and does not produce visible mechanical damage within 100 wrinkling cycles, making the sensing performance consistent after wrinkling (Fig. 10d3 and Fig. 10d4). Kim et al. proposed a wearable smart sensor system attached to the eyeball to detect glucose within tears [200]. In their work, the wearable sensor uses parylene as a flexible substrate and has reliable sensing performance within 10,000 stretching and relaxation cycles. Wang et al. proposed a flexible wearable sensor attached to human tissue or skin surface to detect the TNF-α of sweat (Fig. 10e1–e4) [81]. Here, the wearable sensor uses a polyester film (Mylar) thickness of 2.5um as a flexible substrate. 125% elongation), and has no visible mechanical damage within 500 bending cycles (bending radius of 40 µm), twisting cycles (angles from − 180°to 180°), stretching cycles (stretching the length of 125%) (Fig. 10e5–e7). Yoo et al. proposed an epidermal skin-type point-of-care device to detect prostate cancer antigen (PSA) protein (Fig. 10f1 and f2) [196]. In their work, the device uses polyimide (PI) as a flexible substrate and has the same sensing performance within 10,000 bending cycles (bending radius of 10 mm) (Fig. 10f3 and Fig. 10f4). Huang et al. proposed a wearable biosensor mounted on the eyeball to detect the L-cysteine of tears (Fig. 10g1 and g2) [197]. Here, the biosensor uses transparent PET as a flexible substrate and has no visible mechanical damage within 100 cycles of large deformations (bending at radii 175 µm, folding at 150°, and shrinking at 50%) (Fig. 10g3 and Fig. 10g4).

It is foreseeable that the real-time monitoring wearable device combining FET with wearable technology will be applied to more life scenarios, further promoting the iteration of FET.

### Exploring the biosensing of FET in disease-related biomarkers

2D material-based FET biosensors have made remarkable progress in biosensing, including infectious diseases, genetic diseases, and cancers. By detecting changes in trace molecules such as viruses, bacteria, genes, and cancer biomarkers, these biosensors enable accurate disease diagnosis. As a result, they hold great promise in providing clinicians with more precise tools for diagnosis and treatment.

#### Biosensing: infectious diseases

Infectious diseases are caused by pathogens such as viruses, bacteria, and fungi. To detect these diseases, 2D material-based FET biosensors use fixed antibodies or nucleic acid probes specific to the pathogen or pathogen-related molecule. When the pathogen or pathogen-related molecule enters the biosensor, it binds to the antibody or nucleic acid probe fixed on the surface, changing the sensor output signal. This signal change can be detected by the sensor to determine whether the pathogen or pathogen-related molecule is present in the sample. These biosensors have been widely used in the detection of various infectious diseases, which can be broadly classified into two categories:

1. Viral infection-based diseases: 2D material-based FET biosensors can be used to detect the RNA or DNA of viruses. For example, Sun et al. have used graphene-based FET biosensors to detect the RNA of the novel coronavirus [201]. Ji et al. also developed InSe-based FET biosensors for detecting the coronavirus [202]. Majd et al. have used MoS_2_ nanosheet-based FET biosensors to detect the BRCA1 gene of humans [203]. Besides detecting RNA and DNA, 2D material FET biosensors can detect viral proteins. For instance, Palacio et al. have employed graphene-based FET biosensors to detect the presence of the hepatitis C virus core protein [204]. By measuring the degree of specific binding between the virus DNA/RNA or protein and the biosensor surface, the presence of the virus in the sample can be determined.
2. Bacterial infection-based diseases: 2D material-based FET biosensors can detect bacteria. For instance, Masurkar et al. have successfully utilized MoS_2_-based FET biosensors to detect Escherichia coli, achieving a detection limit of 10 colony-forming units per milliliter (CFU/mL) [205]. Kim et al. have used graphene-based FET biosensors to identify the quantity of Escherichia coli by detecting changes in bacterial shell composition [206]. When Escherichia coli comes into contact with graphene, charge transfer occurs due to the chemical composition on the bacterial surface, resulting in changes in the electrical properties of the graphene film. By measuring the quantity of charge transfer, the concentration of Escherichia coli can be determined in the samples.

#### Biosensing: genetic diseases

2D materials-based FET biosensors have demonstrated promising potential for detecting certain genetic diseases. Single nucleotide polymorphism (SNP) is a common form of genetic variation associated with many diseases' development. 2D materials-based FET biosensors can detect SNP by measuring changes in the charge distribution in the mutated region of a gene. For example, Danielson and colleagues demonstrated the use of graphene-based field-effect transistor (FET) biosensors to distinguish between a target DNA strand and a strand containing a single nucleotide polymorphism [92]. Balderston et al. have used graphene-based FET biosensors to identify single nucleotide mutations in DNA associated with sickle cell anemia [2]. In addition, hemophilia is a genetic disease caused by a deficiency of clotting factors. Schuck et al. utilized graphene-based FET biosensors to detect the concentration of clotting factors in the blood, enabling the detection of hemophilia [207].

#### Biosensing: cancers

2D materials-based FET biosensors have been applied in the diagnosis of various cancers, including the following:

1. Lung cancer: Zhou et al. and Zheng et al. have developed biosensors for detecting lung cancer-related biomarkers using graphene-based and reduced graphene oxide-based sensing materials, respectively. The sensitivity of these biosensors is high, with Zhou et al.'s biosensor capable of detecting carcinoembryonic antigen (CEA) at concentrations as low as 100 pg/mL [208], and Zheng et al.’s biosensor showing a positive linear relationship with the concentration of neuron-specific enolase (NSE) in the range of 0.1 to 2000 ng/mL, with a detection limit of 50 pg/mL [209]. These findings suggest that biosensors utilizing graphene-based materials hold promise for the early detection of lung cancer.
2. Breast cancer: Majd et al. utilized reduced graphene oxide-based FET biosensors to detect the cancer marker CA 125 with a limit of detection (LOD) of 0.5 nU/mL [210]. Similarly, Ji et al. developed InSe-based FET biosensors that exhibit rapid detection capabilities for CA125 protein levels in the blood of breast cancer patients within 20 min, with a detection range spanning from 0.01–1000 U/mL [211].
3. Liver cancer: Kim et al. utilized graphene-based FET biosensors to detect alpha-fetoprotein (AFP), a liver cancer biomarker in the blood of liver cancer patients [212]. In their study, the biosensor successfully detected AFP in the plasma of liver cancer patients at a concentration of 12.9 ng/mL.

While these applications are currently in the experimental phase, the continuous advancement of 2D material-based FET biosensor technology suggests that they will play an increasingly crucial role in medical diagnosis and treatment in the future.

## Outlook and current lacuna in 2D material-based FET biosensing

The 2D material-based FET biosensor is a novel type of transistor that exhibits excellent electrical properties such as high carrier mobility and low noise. Here, the current lacuna and outlook of 2D material-based FET biosensors are reviewed from the following aspects:

Focusing on research direction 1, numerous sensing mechanisms for 2D material-based FET biosensors have been proposed in the past decade. However, a universal sensing mechanism has yet to be identified, which is the main obstacle for the field of FET biosensors to overcome. Going forward, it is crucial to explore a universal sensing mechanism that can account for all experimental results.

Focusing on research direction 2, a range of response signals have been explored as indicators for detecting target molecules. Progress has been made in understanding the relationship between response signals and sensing mechanisms. However, researchers tend to focus on a single type of response signal for biosensing, potentially overlooking more sensitive detection methods using other response signals. To address this, in the future, researchers should select the most appropriate type of response signal for indicating target molecule detection and also actively explore new types of response signals to broaden their detection capabilities.

Focusing on research direction 3, optimization strategies for sensing materials, probe immobilization methods, probe types, and multiplying target signals methods have been extensively studied. Regarding sensing materials, researchers can choose the type of material suitable for their needs based on their experimental demands, preparation process, and cost considerations. Various probe immobilization methods and probe types have been investigated to enhance sensing performance. Multiplying target signal methods have been explored to improve the response performance of target biomolecules. There are promising methods to improve recognition efficiency, such as combining a PMO probe with a Y-shaped or tetrahedral structure. Moving forward, the CRISPR-Cas system combined with FET holds great potential for achieving a breakthrough in detection sensitivity.

Focusing on research direction 4, Iterative strategies combining FET with microfluidic, microelectronics, and wearable technologies have been proposed. These strategies are expected to drive the development of FET. A portable smart-sensing device combining microfluidic and microelectronics technology with FET has excellent potential in on-site detection. Additionally, real-time monitoring wearable devices that integrate FET with wearable technology are expected to expand the application scenarios of FET and further promote its iteration.

Overall, although 2D material-based FET biosensors currently have some limitations, ongoing technological advancements will gradually refine and improve these shortcomings, allowing for the increasingly widespread development and application of 2D material-based FET biosensors in the field of biosensing.

## Conclusions

Focusing on the multidisciplinary technical details of FET biosensors, we first summarized a series of existing breakthroughs and dialectical evaluations of four research directions. Aiming at each research direction, we pointed out the promising technology and prospects to promote the researchers to quickly and comprehensively understand the field.

- Direction 1: Exploring the sensing mechanism of FET to detect biomolecules: It is necessary to explore a universal sensing mechanism to explain all experimental results.
- Direction 2: Broadening response signal types of FET: Researchers should select the optimal response signal type to determine detection results according to the application scenario.
- Direction 3: Optimizing the sensing performance of FET: The detection potential of FET is further exploited by adopting an optimization strategy from multiple aspects (the sensing material, the probe immobilization method, the probe type, and so on).
- Direction 4: Driving the iterative strategy of FET: Wearable smart devices integrating on-site detection and real-time monitoring functions will create a new technological revolution by combining microelectronics and wearable technology with FET.

Finally, we believe that the FET has great potential and bright prospects in the field of biomolecular detection!

## Data Availability

Not applicable.
